# Thioxobimanes

**DOI:** 10.1021/acs.joc.3c00873

**Published:** 2023-09-15

**Authors:** Partha Jyoti Das, Ankana Roy, Ashim Nandi, Ishita Neogi, Yael Diskin-Posner, Vered Marks, Iddo Pinkas, Sara Amer, Sebastian Kozuch, Michael Firer, Michael Montag, Flavio Grynszpan

**Affiliations:** † Department of Chemical Sciences, Ariel University, Ariel 40700, Israel; ‡ Department of Chemistry, Ben-Gurion University, Beer Sheva 841051, Israel; § Department of Chemical Research Support, Weizmann Institute of Science, Rehovot 76100, Israel; ∥ Department of Chemical Engineering and Biotechnology, Ariel University, Ariel 40700, Israel

## Abstract

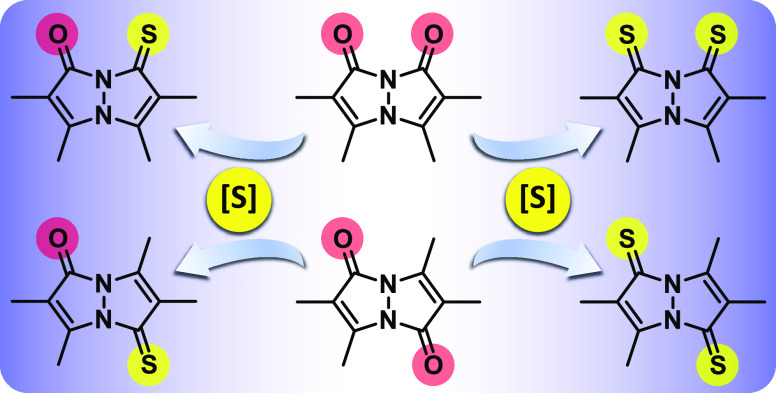

Dioxobimanes, colloquially known as bimanes, are a well-established family of *N*-heterobicyclic compounds that share a characteristic core structure, 1,5-diazabicyclo[3.3.0]octadienedione, bearing two endocyclic carbonyl groups. By sequentially thionating these carbonyls in the *syn* and *anti* isomers of the known (Me,Me)dioxobimane, we were able to synthesize a series of thioxobimanes, representing the first heavy-chalcogenide bimane variants. These new compounds were extensively characterized spectroscopically and crystallographically, and their aromaticity was probed computationally. Their potential role as ligands for transition metals was demonstrated by synthesizing a representative gold(I)–thioxobimane complex.

## 1 Introduction

**Figure 1:**
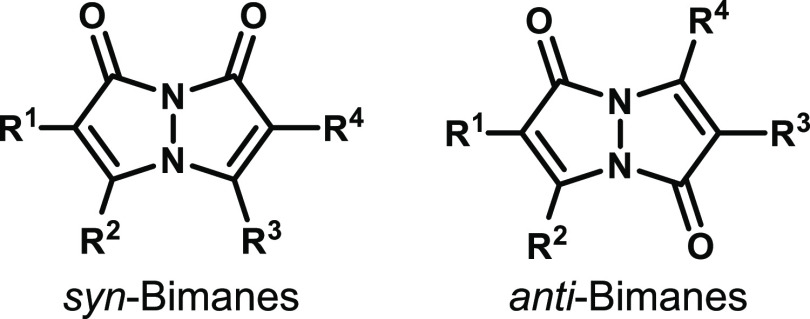
General molecular structures of bimanes (R^1–4^ = organic or inorganic substituents).

Dioxobimanes, also known simply as bimanes, are *N*-heterobicyclic compounds that share a common core structure, 1,5-diazabicyclo[3.3.0]octadienedione, as presented in Figure 1. They were introduced in the late 1970s by Kosower and co-workers,^1^ who established their synthetic protocols,^2^ and investigated the spectroscopic properties,^3,4^ molecular structures,^5^ and many other aspects of this family of materials. More recently, we have reported an alternative synthetic procedure for such compounds, enabling their convenient large-scale preparation in moderate-to-good yields from commercially available starting materials.^6^

For a given bimane, two constitutional isomers can typically be isolated from its synthetic reaction mixture, namely, *syn* and *anti* (Figure 1). The *syn* isomer, which is typically the major product, is polar and usually exhibits strong fluorescence in the ultraviolet–visible (UV–vis) range. The *anti* isomer, which is normally the minor product, is typically much less polar and nonfluorescent. The photophysical properties of bimanes can be modulated by chemical modifications of the bicyclic bimane core through the installment of various electron-withdrawing or -donating substituents (R^1–4^ in Figure 1). This is of particular interest in the case of *syn*-bimanes, which have found use as fluorescent labels, primarily for biological applications.^7^ These small fluorophores have been highly instrumental in the development of biochemical methodologies, mainly those involving the detection and analysis of sulfhydryl species, such as H_2_S and glutathione, both in vitro and in vivo.^8^ They have also made notable contributions to a variety of biologically related disciplines like enzymology,^9^ pharmacology,^10^ toxicology,^11^ and environmental studies^12^ and have also been applied, among other things, in fundamental investigations of photophysical processes^13^ and even as photolabile protecting groups.^14^

**Figure 2:**
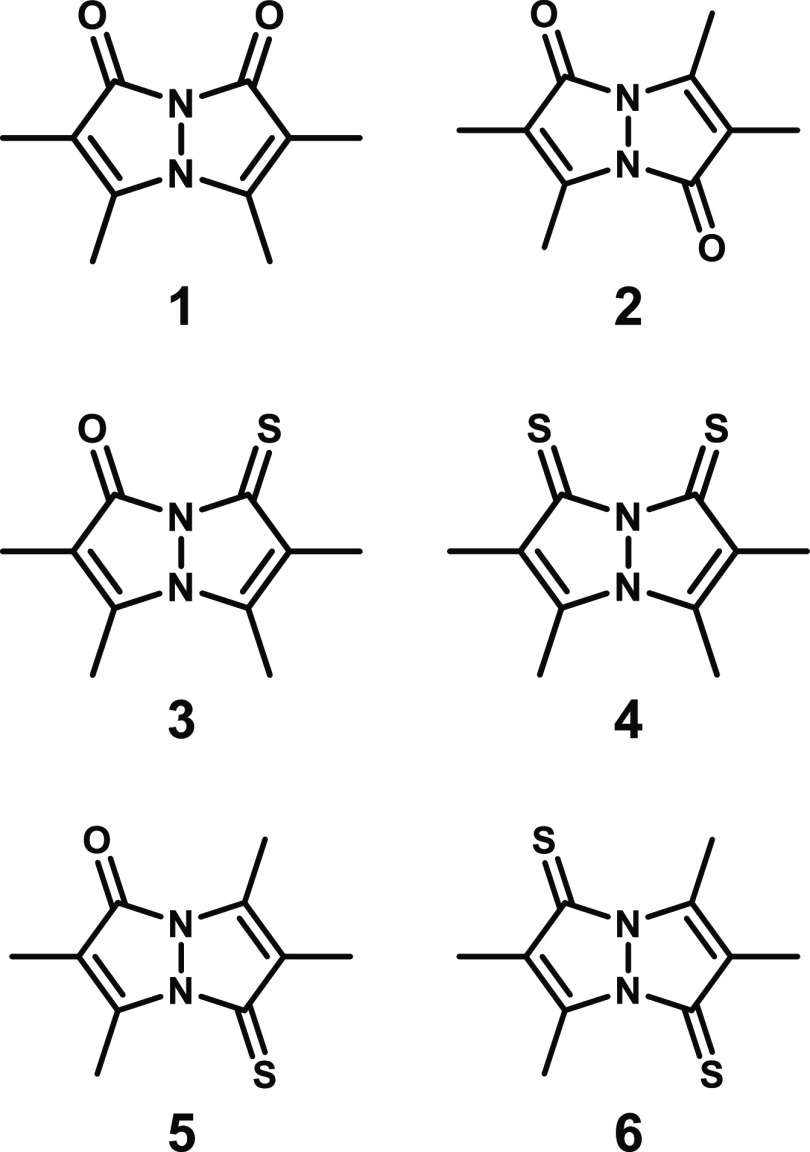
Structures of *syn*-(Me,Me)dioxobimane (**1**), *anti*-(Me,Me)dioxobimane (**2**), and their thionated derivatives.

Several years ago, we described the first example of metal-bimane coordination in the form of a cationic Pd(II) complex containing the previously reported *syn*-(Me,Me)dioxobimane [*syn*-(O,O)bimane, **1**; Figure 2]^1,2^ as an O-donor chelating ligand.^15^ Subsequently, we also studied the interaction of **1** with Li(I) and Na(I) and showed that this bimane exhibits several coordination modes, forming both discrete and polymeric coordination architectures.^16,17^ Moreover, we demonstrated that sodium coordination to **1** induces fluorescence quenching in solution,^16^ and that this bimane can be used for the highly sensitive fluorescence-based detection of trace Co(II).^18^ More recently, we reported the use of a triazole-functionalized *syn*-bimane as a core component of highly sensitive fluorescent chemosensors for iodine^19^ and water.^20^

In order to access new bimane derivatives with different photophysical and coordination characteristics, while avoiding significant variations in the basic bimane structure, we reasoned that the carbonyl oxygen atoms of the original dioxobimanes could be replaced by the heavier congener sulfur, which is softer and less electronegative than oxygen. Thus, we applied traditional thionation chemistry, using Lawesson’s reagent and P_4_S_10_, to convert the known *syn*- and *anti*-(O,O)bimane (**1** and **2**, respectively)^1,2^ into their corresponding mono- and dithionated derivatives, namely, *syn*-(Me,Me)oxothioxobimane [*syn*-(O,S)bimane, **3**], *syn*-(Me,Me)dithioxobimane [*syn*-(S,S)bimane, **4**], *anti-*(Me,Me)oxothioxobimane [*anti*-(O,S)bimane, **5**], and *anti*-(Me,Me)dithioxobimane [*anti*-(S,S)bimane, **6**], all of which are depicted in Figure 2. Herein, we present a detailed account of these new members of the bimane family, encompassing their preparation, spectroscopic and crystallographic properties, and computational analysis of their aromaticity. Furthermore, we describe a gold(I) complex of thioxobimane **3** to demonstrate the ability of these new compounds to serve as ligands for transition metals.

## 2 Results and Discussion

### 2.1 Synthesis of the Thioxobimanes

**Scheme 1:**
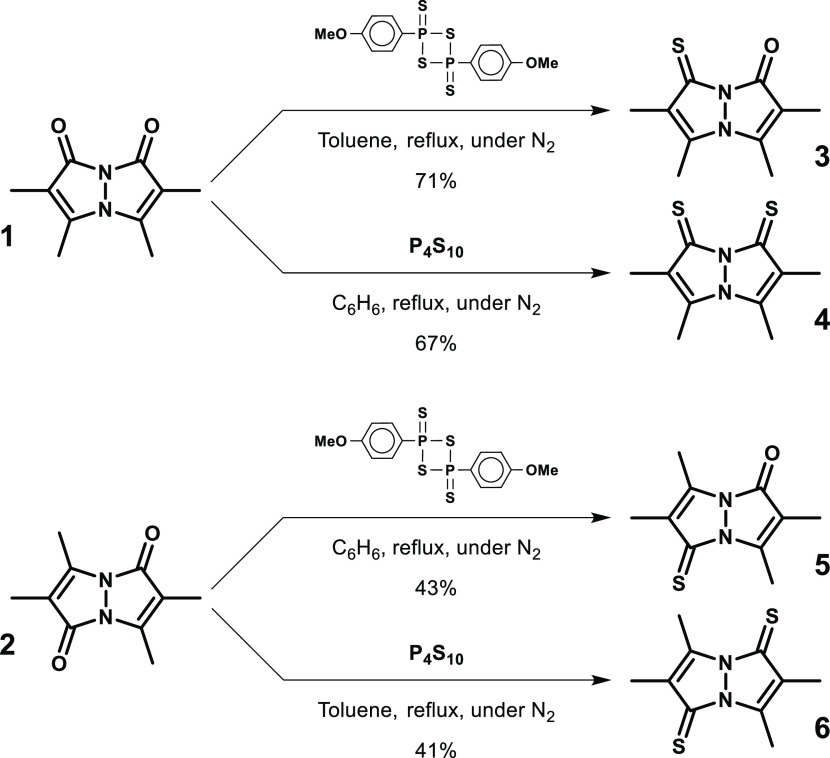
Synthesis of the Thioxobimanes from the Parent Dioxobimanes

The use of Lawesson’s reagent is a mild and convenient way to thionate carbonyl compounds, such as ketones, esters, and amides, transforming the carbonyl moiety into thiocarbonyl.^21^ We sought to utilize this reaction to convert **1** and **2** into the corresponding thioxobimanes, and it indeed proved to be satisfactory for monothionations, as shown in Scheme 1. Thus, reaction of *syn*-(O,O)bimane **1** with 0.6 equiv of Lawesson’s reagent in refluxing toluene afforded *syn*-(O,S)bimane **3** in 71% isolated yield. Similarly, reaction of **2** with 0.8 equiv of Lawesson’s reagent in refluxing benzene gave *anti*-(O,S)bimane **5** in 43% isolated yield. Generation of the corresponding dithioxobimanes was realized using P_4_S_10_ instead of Lawesson’s reagent (Scheme 1). Thus, *syn*-(S,S)bimane **4** was synthesized in 67% isolated yield by treating **1** with 1.1 equiv of P_4_S_10_ in refluxing benzene, whereas *anti*-(S,S)bimane **6** was prepared in 41% isolated yield through the reaction of **2** with 1.5 equiv of P_4_S_10_ in refluxing toluene.

The abovementioned synthetic procedures highlight the thermal stability of the dioxobimanes and their thionated derivatives. Kosower and co-workers have demonstrated that *syn*-bimane **1** decomposes appreciably only at high temperatures, exhibiting a half-life of approximately 10 h at 280 °C, under an anaerobic atmosphere.^2^ Under these conditions, **1** converted mainly into *anti*-bimane **2**, which is the thermodynamically more stable isomer. Our synthetic procedures were much milder, and the highest temperature to which the bimanes were exposed was that of refluxing toluene, i.e., ∼110 °C. When **1** was subjected to these conditions for 1 h, no decomposition nor discernible amounts of **2** were observed, as judged by both HPLC and ^1^H NMR spectroscopy. Moreover, all thionated bimanes were obtained in significant yields despite being exposed to heating in either refluxing toluene or benzene.

### 2.2 UV–Vis Absorption and Fluorescence of the Thioxobimanes

**Figure 3:**
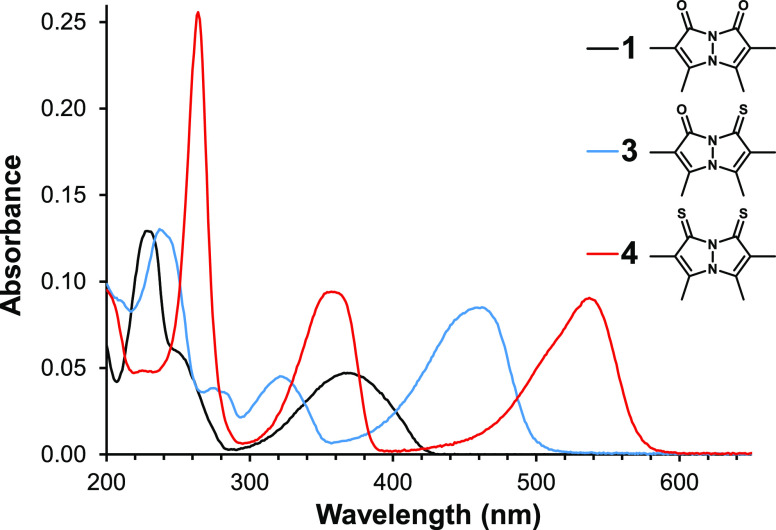
UV–vis absorbance spectra of *syn*-bimanes **1**, **3**, and **4** in CH_3_CN. Each solution contained 10 μM of the respective bimane.

**Figure 4:**
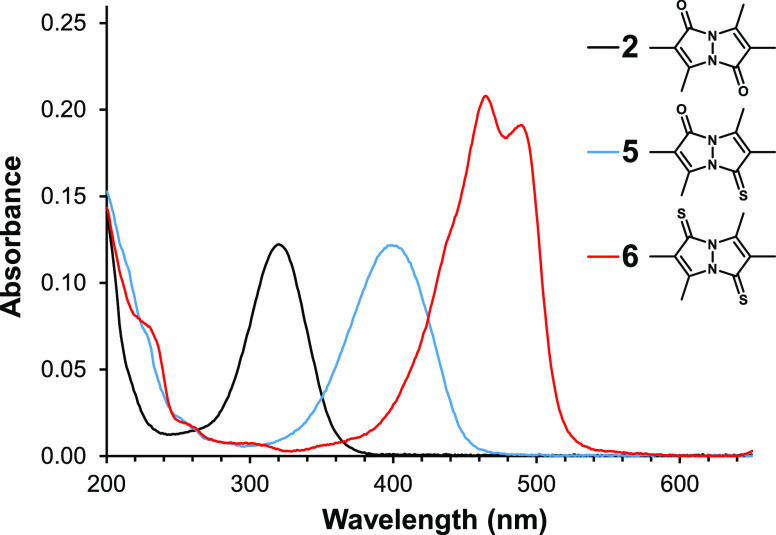
UV–vis absorbance spectra of *anti*-bimanes **2**, **5**, and **6** in CH_3_CN. Each solution contained 10 μM of the respective bimane.

Visual inspection of solid samples of bimanes **1**, **2**, and their thionated congeners clearly reveals the impact of replacing oxygen by sulfur on the color of the bimanes. Whereas *syn*-(O,O)bimane **1** is light yellow, **3** is orange and **4** is purple, and while *anti*-(O,O)bimane **2** is colorless, **5** is yellow and **6** is red. As can be seen in Figures 3 and 4, which depict the UV–vis absorbance spectra of these bimanes in acetonitrile, the observed color changes reflect marked redshifts in the absorption of the bimane chromophore upon successive substitution of S for O.

In the spectral range of 200–800 nm, an acetonitrile solution of *syn*-(O,O)bimane **1** exhibits two distinct absorption peaks at 228 and 368 nm, as well as a shoulder at ∼253 nm. Its monothionated derivative **3** displays four separate absorption bands at 238, 274, 322, and 462 nm, and its dithionated variant **4** exhibits three bands at 264, 357, and 535 nm. For each of these bimanes, the shortest-wavelength peak is also the most intense. The corresponding *anti* isomers—**2**, **5**, and **6**—give rise to much simpler UV–vis absorption spectra, containing only one peak in the case of **2** and **5**—at 320 and 400 nm, respectively—and two overlapping peaks in the case of **6**, at 465 and 489 nm. It should be noted that the dithioxobimanes, **4** and **6**, generally exhibit more intense absorption peaks than their dioxo and oxothioxo congeners. Table 1 compiles the wavelengths and extinction coefficients at the absorption maxima of bimanes **1**–**6** in CH_3_CN.

**Table 1:** Wavelengths at Maximal Absorption (λ_max_) and the Corresponding Extinction Coefficients (ε) for the Dioxobimanes, Oxothioxobimanes, and Dithioxobimanes in Acetonitrile

bimane	λ_max_ (nm)	ε (10^3^cm^–^^1^ M^–^^1^)
**1**	228	12.67(6)
	368	4.77(2)
**2**	320	11.9(2)
**3**	238	12.0(2)
	274	3.47(7)
	322	4.26(9)
	462	8.2(2)
**4**	264	25.6(1)
	357	9.48(2)
	535	9.11(2)
**5**	400	13.42(7)
**6**	465	21.6(2)
	489	19.9(2)

Thionation-induced bathochromic shifts are well-documented for carbonyl-containing chromophores, such as those of coumarin,^22^ naphthalene diimide (NDI),^23^ and diketopyrrolopyrrole (DPP)^24^ dyes. This is a manifestation of the decrease in the energy gap between the occupied and unoccupied frontier orbitals of such chromophores, which occurs when oxygen is replaced by the heavier sulfur.^25,26^ For the parent dioxobimanes **1** and **2**, density functional theory (DFT) calculations have previously demonstrated that the electronic transitions between those frontier orbitals, which fall within the UV–vis range, are π → π* and n → π*.^27,28^ Moreover, the lowest energy transitions in these dioxobimanes have been shown to be π → π*, and these are associated with the observed absorption peaks of longest wavelength. Thiocarbonyl-containing chromophores also exhibit π → π* and n → π* transitions between their frontier orbitals, but here the lowest energy transitions are typically n → π*,^26,29^ and this may well be the case for thioxobimanes **3**–**6**. However, these transitions are symmetry-forbidden and hence weak and likely to be unobservable. Thus, the longest-wavelength peaks observed for **3**–**6**, which are relatively strong, are most probably due to π → π* transitions.

Although all absorption peaks of **1** and **2** exhibit redshift upon successive substitution of S for O, the most significant effects involve the longest-wavelength peaks. In the *syn*-bimane series, this redshift amounts to 94 nm for **1** → **3**, and an additional 73 nm for **3** → **4**. For the *anti*-bimanes, the redshift for **2** → **5** is smaller, at 80 nm, but for **5** → **6** it is larger, amounting to 89 nm. Interestingly, the overall redshift observed upon going from the dioxo- to the dithioxobimane is nearly identical for the *syn* and *anti* isomers, at 167 and 169 nm, respectively. These observations clearly indicate that the π → π* transitions responsible for these peaks involve orbitals with strong chalcogen contributions.

It should be noted that the absorbance spectrum of the *syn*-(O,S)bimane **3** is not a linear combination of the corresponding spectra of *syn*-(O,O)bimane **1** and *syn*-(S,S)bimane **4**, and the same is true of the respective *anti*-bimanes. This indicates that the two five-membered rings comprising the bimane core do not behave as two independent chromophores but instead constitute one intact bicyclic chromophore.

As mentioned above, *syn*-dioxobimanes are generally known for their bright fluorescence. This is clearly apparent in the case of bimane **1**, which emits in the blue region, and has been shown by Kosower and co-workers to exhibit high quantum yields over a wide range of solvent polarities, i.e., from 0.9 in dioxane (λ_em_ ≈ 420 nm) to 0.5 in a 93:7 (v/v) water/dioxane mixture (λ_em_ ≈ 480 nm).^4^ By contrast, we found its thionated variants, **3** and **4**, to display no observable fluorescence in the UV–vis range, either in CH_3_CN or CHCl_3_.^30^ Such thionation-induced fluorescence quenching has been reported for a variety of other carbonyl-containing fluorophores.^31−34^ In the case of the *anti* isomers, the parent dioxobimane **2** is inherently nonfluorescent,^28^ as are its thionated variants **5** and **6**.^30^

The fact that dioxobimane **1** exhibits strong visible fluorescence, whereas the thionated compounds **3** and **4** do not, raises the prospect of utilizing *syn*-thioxobimanes as turn-on fluorescent probes for oxidants that are capable of converting them into the parent dioxobimanes. Such oxidant-induced thiocarbonyl-to-carbonyl transformations have been employed, for example, in thiocoumarin-based fluorescent chemosensors for hypochlorite.^35^ Preliminary results indicate that treating thioxobimane **4** with either hypochlorite or hydrogen peroxide restores fluorescence (see Supporting Information), thereby laying the basis for further studies along this direction.

### 2.3 Vibrational Spectroscopy

**Figure 5:**
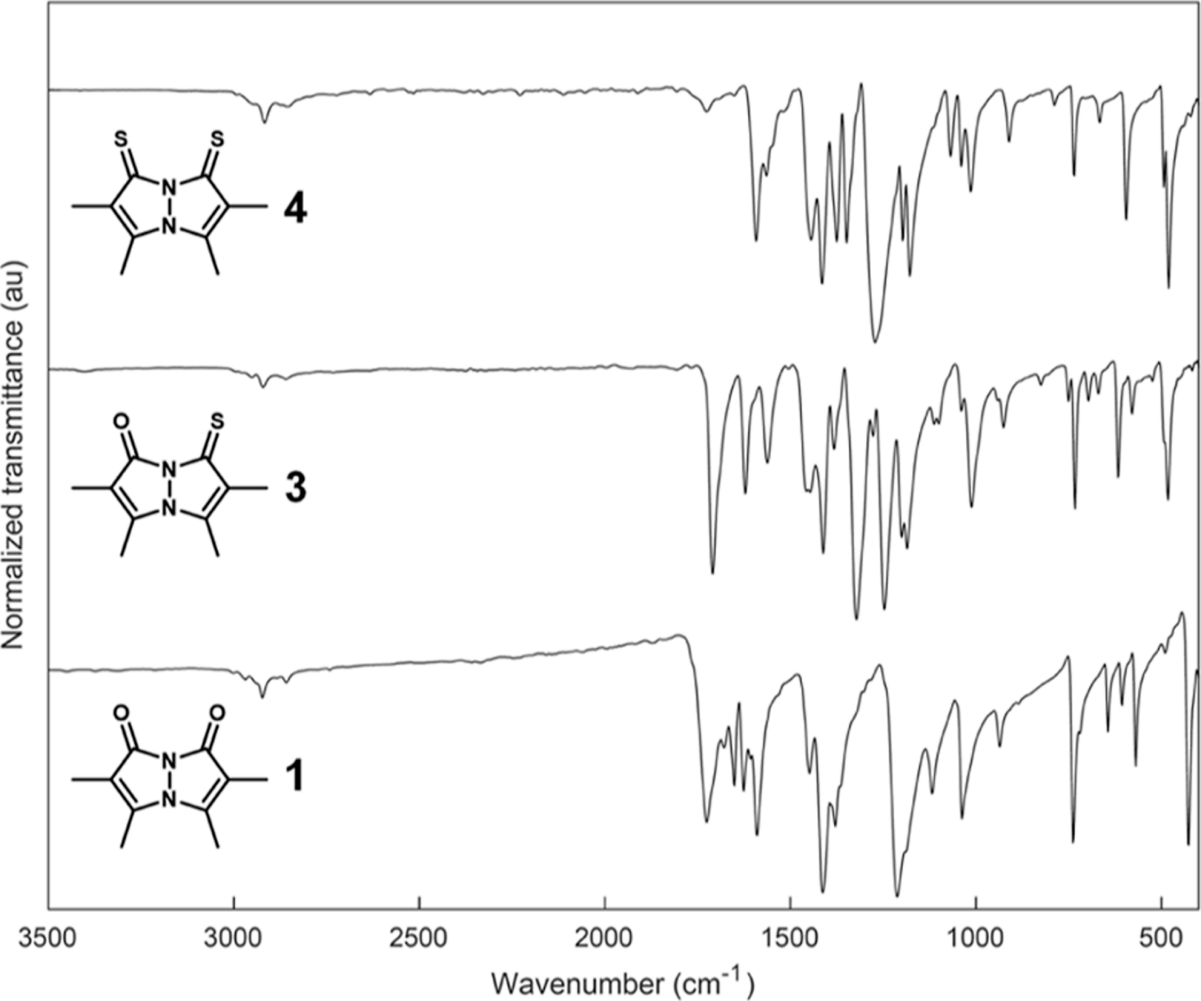
ATR-IR spectra of crystalline *syn*-bimanes **1**, **3**, and **4**. Each spectrum was normalized to its strongest-intensity peak.

**Figure 6:**
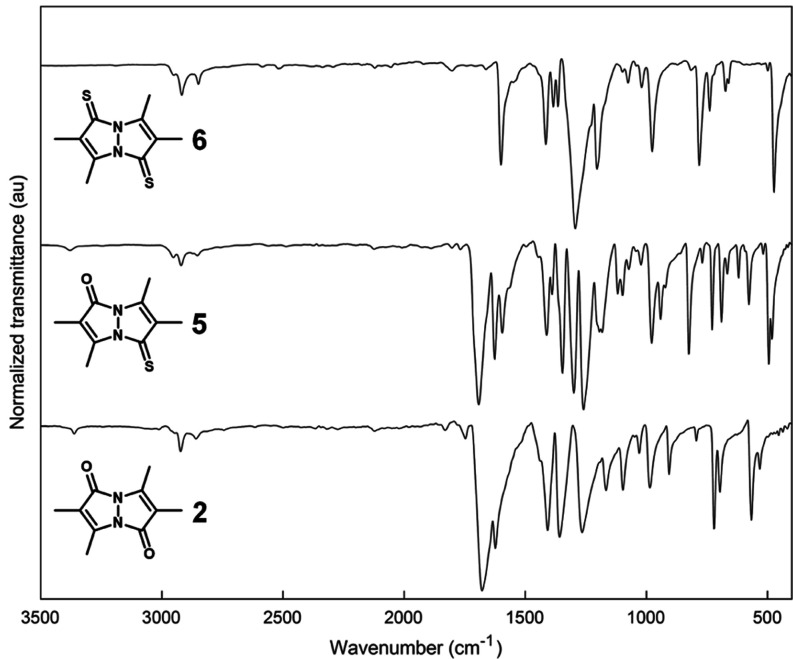
ATR-IR spectra of crystalline *anti*-bimanes **2**, **5**, and **6**. Each spectrum was normalized to its strongest-intensity peak.

**Figure 7:**
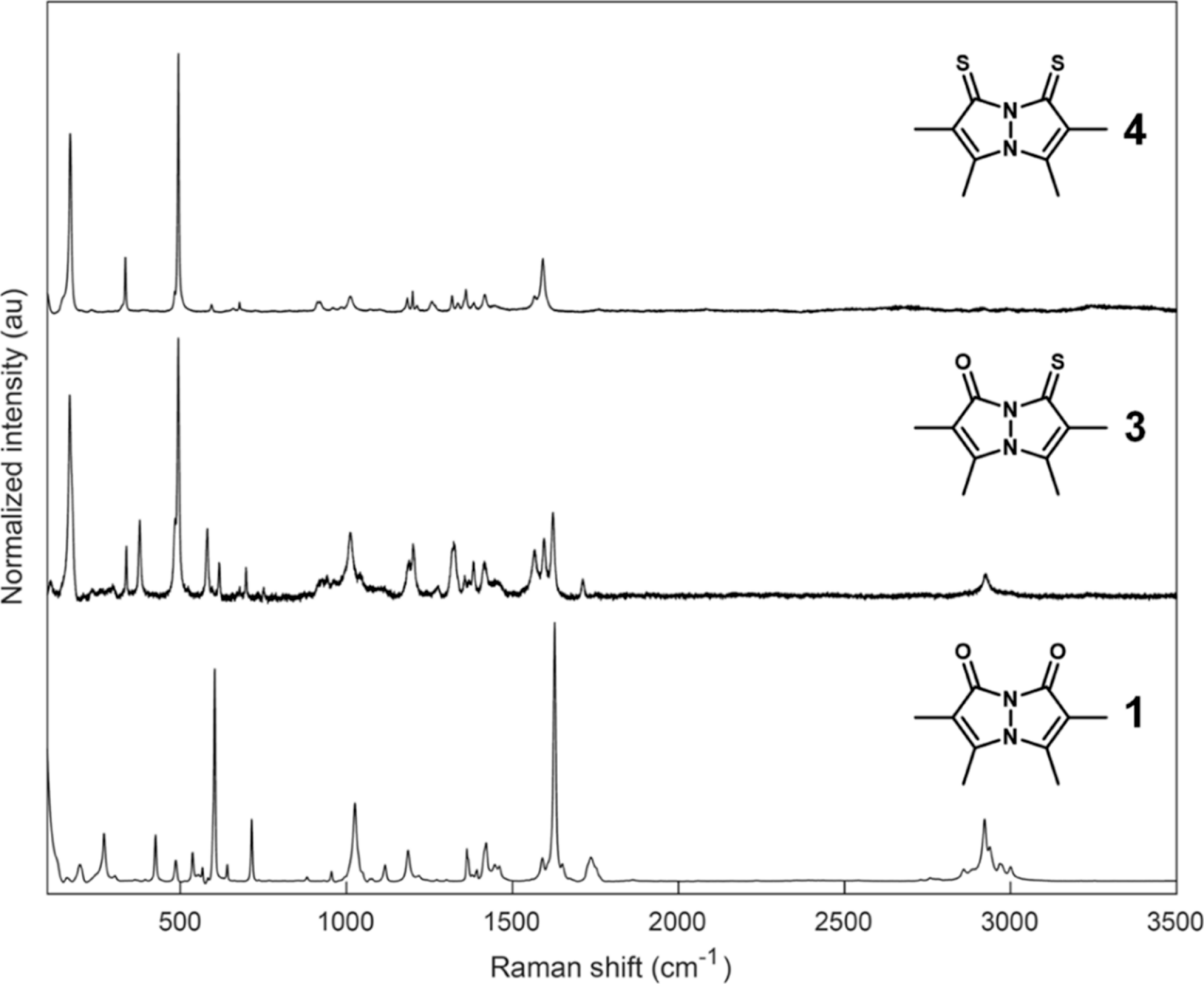
Raman spectra of crystalline *syn*-bimanes **1**, **3**, and **4**. Each spectrum was normalized to its strongest-intensity peak.

**Figure 8:**
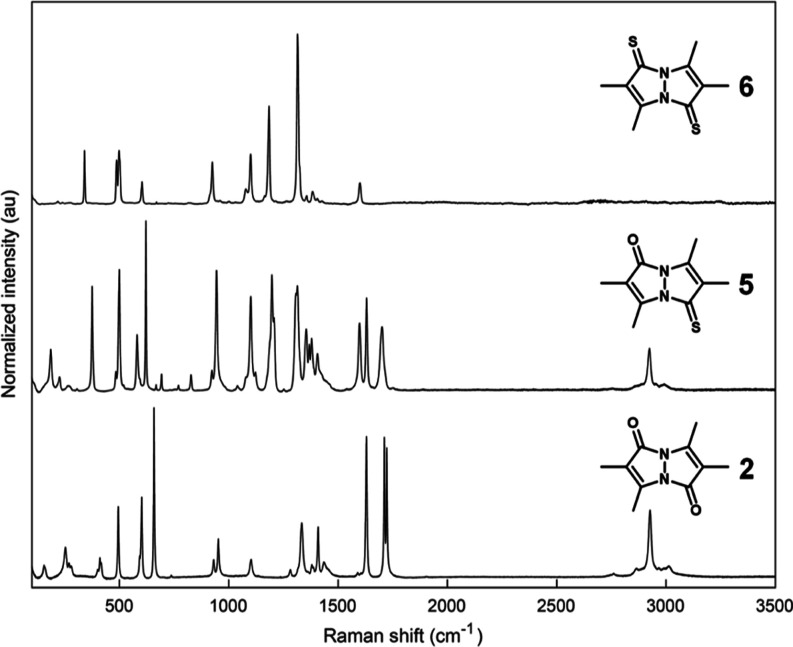
Raman spectra of crystalline *anti*-bimanes **2**, **5**, and **6**. Each spectrum was normalized to its strongest-intensity peak.

The solid-state infrared (IR) spectra of *syn*-bimanes **1**, **3**, and **4**, recorded in the attenuated total reflectance (ATR) mode, are presented in Figure 5, and those of *anti*-bimanes **2**, **5**, and **6** are presented in Figure 6. The respective Raman spectra are shown in Figures 7 and 8. Assignment of the vibrational bands is based on a comparison between the various *syn*- and *anti*- bimanes, assisted by their calculated IR spectra (see Supporting Information),^36^ and assessment of the corresponding simulated vibrational modes. According to our calculations, many of the observed bands are the result of complex combination modes, and only those identified with a high degree of certainty are mentioned here.

As can be inferred from the IR data, all bimanes exhibit 3–5 weak bands at 2830–3000 cm^–1^, attributable to C–H stretching modes, with no significant variations being observed between the various bimanes. The Raman spectra of the dioxo- and oxothioxobimanes also display medium-to-weak bands at similar frequencies, but, interestingly, the dithioxobimanes lack observable C–H stretching bands. The strong IR peaks associated with the carbonyl stretching vibrations of *syn*-(O,O)bimane **1** appear at 1726 (*sym*) and 1651 cm^–1^ (*asym*), whereas that of *syn*-(O,S)bimane **3** appears at 1709 cm^–1^. All of the respective Raman bands are weak. In the case of *anti*-(O,O)bimane **2**, only the asymmetric C=O stretch is observable in the IR spectrum, at 1678 cm^–1^, and the corresponding peak for *anti*-(O,S)bimane **5** appears at 1691 cm^–1^. In the respective Raman spectra, **5** gives rise to a strong band at about the same frequency, whereas **2** only exhibits the symmetric C=O stretching band as a strong peak at 1723 cm^–1^.

Adjacent to the IR carbonyl peaks lie the vibrational bands associated with C=C stretching. Dioxobimane **1** displays two such bands at 1626 (*sym*) and 1589 cm^–1^ (*asym*), whereas dithioxobimane **4** displays the respective peaks at 1593 and 1564 cm^–1^. *syn*-(O,S)bimane **3** seems to combine the characteristic C=C vibrational bands of **1** and **4**, exhibiting peaks at 1622 and 1562 cm^–1^, corresponding to the **C**=**C**–CO (carbonyl-conjugated) and **C**=**C–**CS (thiocarbonyl-conjugated) bonds, respectively. An analogous picture emerged from the IR spectra of the *anti*-bimanes. Thus, the **C**=**C**–CO peaks for *anti*-(O,O)bimane **2** and *anti*-(O,S)bimane **5** appear at nearly the same frequency—1624 and 1626 cm^–1^, respectively—which is also practically identical to that of the corresponding *syn*-bimanes. The **C**=**C**–CS bands of **5** and *anti*-(S,S)bimane **6** appear at 1595 and 1601 cm^–1^, respectively. All of these C=C stretching bands have also been identified in the corresponding Raman spectra.

In the fingerprint region, the IR spectra of the three *syn*-bimanes exhibit several noteworthy features. First, they all share similarly positioned strong-to-medium bands in the ranges 1370–1455, 1160–1205, 1030–1050, and 725–745 cm^–1^. Among these, the highest frequency peaks involve both methyl C–H deformations and enamine C–N stretching modes, as suggested by our calculations. Second, within the spectral window 1230–1360 cm^–1^, where bimane **1** is largely IR-transparent, its thionated derivatives display prominent peaks, which are shown by our calculations to arise from thioamide C–N vibrations and C–H deformations, as well as various combination bands. Thus, the most intense bands in the IR spectrum of **3** appear within this window, at 1321 (Me**C–N** + S**C–C**, **C–H**) and 1248 cm^–1^ (O**C–N** + O**C–C**, **C–H**), alongside a medium peak at 1277 cm^–1^ (S**C–N**, **C–H**). Similarly, the strongest band of **4** is observed at 1271 cm^–1^ (S**C–N**, **C–H**) and is accompanied by a medium peak at 1348 cm^–1^ (**N–N** + **C**=**S**, **C–H**). Bimane **1** exhibits one of its strongest absorbance peaks just outside this window, at 1211 cm^–1^ (O**C–N**, **C–H**). None of these vibrational bands are visible in the Raman spectra, except for the aforementioned peak associated with Me**C–N** and S**C–C** stretching in **3**. The IR spectra of the three *anti*-bimanes also exhibit fingerprint bands that are common to all three of these bimanes, but their number is much smaller than for the *syn*-bimanes. Thus, **2**, **5**, and **6** share only three similarly positioned bands at 1400–1430, 1230–1370, and 960–1060 cm^–1^. As was found for the *syn*-bimanes, the medium bands around 1400 cm^–1^ are attributed to enamine C–N stretching and C–H deformations, whereas the strong-to-medium bands between 1230 and 1370 cm^–1^ are assigned to thioamide C–N vibrations, C–H deformations, and combination bands. It is important to note that the C=S stretching vibrations of the thioxobimanes are expected to afford relatively weak IR bands in the fingerprint region, and to be strongly coupled to C–N vibrations,^37^ thereby making them difficult to identify in the respective IR spectra. Thus, of the four thioxobimanes, only in the case of **6** were we able to identify the vibrational band of the thiocarbonyl moiety, as a medium peak at 1203 cm^–1^ (*asym*). This band is not visible in the corresponding Raman spectrum.

### 2.4 NMR Spectroscopy

**Figure 9:**
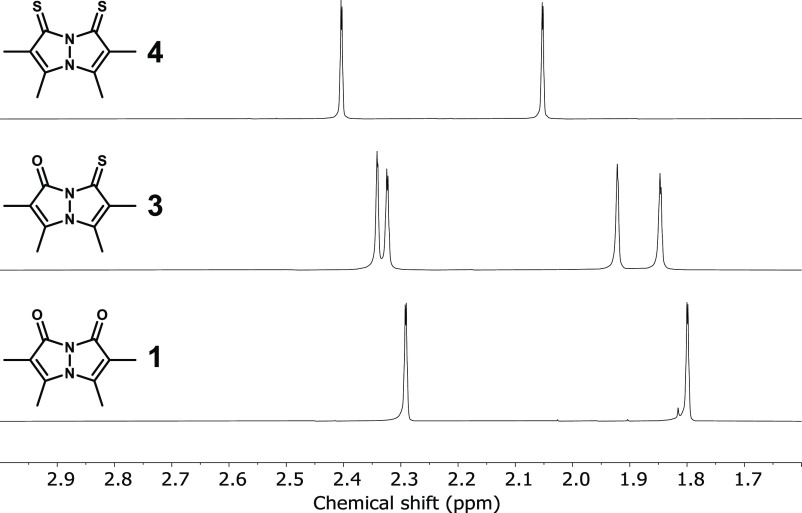
^1^H NMR spectra (400 MHz) of *syn*-bimanes **1**, **3**, and **4** in CDCl_3_.

**Figure 10:**
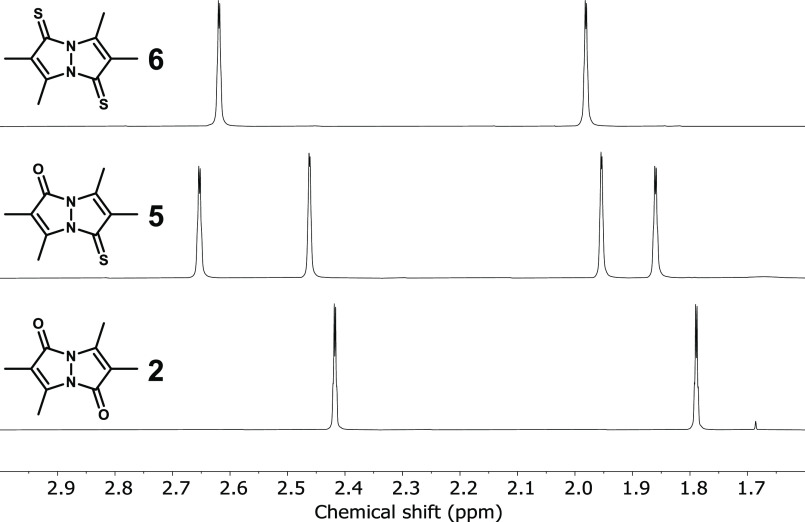
^1^H NMR spectra (400 MHz) of *anti*-bimanes **2**, **5**, and **6** in CDCl_3_.

**Figure 11:**
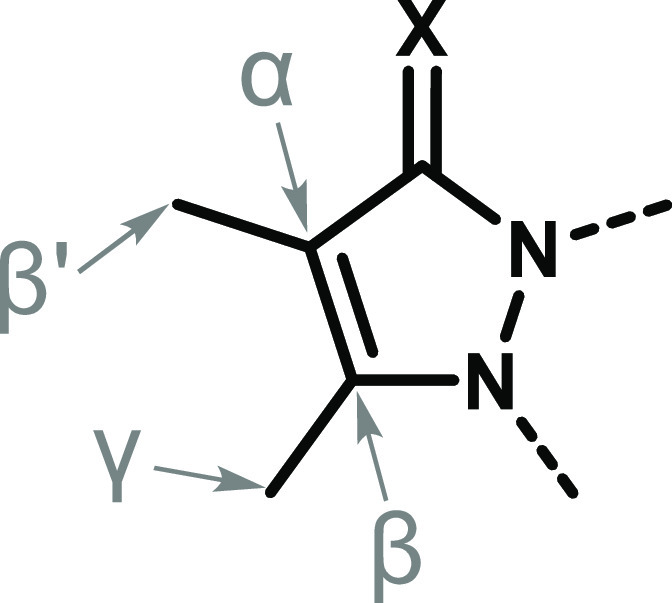
Enumeration scheme for the atomic positions in a pyrazolinone (X = O) or pyrazolinthione (X = S) ring comprising the bimane scaffold.

The ^1^H NMR spectra of bimanes **1**–**6** in CDCl_3_ are depicted in Figures 9 and 10, and their ^1^H and ^13^C chemical shift data are compiled in Table 2 (see Supporting Information for the ^13^C{^1^H} NMR spectra). To assist the discussion, here and elsewhere, the atomic positions in the pyrazolinone or pyrazolinthione rings comprising the bimane scaffolds are designated as shown in Figure 11. As previously reported, the ^1^H NMR spectra of the parent *syn*- and *anti*-dioxobimane, **1** and **2**, each comprise two methyl group resonances with a 1:1 integral ratio, and the same is observed for the new dithioxobimanes **4** and **6**, which belong to the same symmetry point groups as their parent bimanes (*C*_2*v*_ and *C*_2*h*_, respectively, assuming planarity). The mixed oxothioxo variants, **3** and **5**, are of lower symmetry (*C*_s_, assuming planarity), and each exhibits four methyl resonances with a 1:1:1:1 integral ratio. In all cases, the CH_3_ peak associated with the γ position of the pyrazolinone or pyrazolinthione ring is downfield of the peak belonging to the β′ position of the same ring. Moreover, all methyl resonances appear as poorly resolved quartets, reflecting weak 5-bond ^1^H–^1^H coupling between vicinal CH_3_ groups. In the ^13^C{^1^H} NMR spectra of all six bimanes, the resonances associated with the various carbon atom positions are arranged in the same chemical shift order, namely, (thio)carbonyl > β > α > γ > β′.

**Table 2:** Comparison of ^1^H and ^13^C NMR Chemical Shift Data (ppm) for Bimanes **1**–**6** in CDCl_3_

nucleus	position^a^	*syn*-bimanes			*anti*-bimanes		
		1	3	4	2	5	6
^1^H	β′_O_	1.80	1.85		1.79	1.86	
	β′_S_		1.93	2.05		1.95	1.99
	γ_O_	2.29	2.32		2.42	2.65	
	γ_S_		2.34	2.40		2.45	2.63
^13^C	C=O	160.9	159.4		161.7	158.9	
	C=S		177.5	176.0		178.2	175.7
	α_O_	112.6	112.1		112.3	113.1	
	α_S_		124.7	124.5		125.0	125.2
	β_O_	146.0	141.8		145.8	146.2	
	β_S_		141.2	136.5		140.8	141.5
	β′_O_	6.9	6.8		6.5	6.4	
	β′_S_		8.3	8.7		8.3	8.3
	γ_O_	12.0	11.4		10.9	12.8	
	γ_S_		11.4	11.0		10.1	12.4

a Subscripted “O” refers to the pyrazolinone ring; subscripted “S” refers to the pyrazolinthione ring.

A perusal of the NMR data measured for the six bimanes reveals noticeable variations as dioxobimanes **1** and **2** are thionated. In both cases, the ^1^H NMR methyl resonances undergo downfield shifts upon going from (O,O)bimane to (S,S)bimane, amounting to 0.11–0.25 ppm. In the ^13^C{^1^H} NMR spectra, this transformation has the strongest effect on the carbonyl carbon atoms, as would be expected, with downfield shifts of 14.0–15.1 ppm upon replacing both O atoms with S. The next in line to be affected are the olefinic carbon atoms, with the α positions being deshielded by 11.9–12.9 ppm and the β positions being shielded by 4.3–9.5 ppm. Finally, the least-affected carbon nuclei are those of the methyl groups, which are shifted downfield by less than 2.0 ppm, except for the γ-CH_3_ groups of **1**, which are shielded by 1.0 ppm upon dithionation. As mentioned above, **3** and **5** are of reduced symmetry, and they exhibit twice as many ^1^H and ^13^C NMR signals, which are subdivided according to the type of five-membered ring to which they belong, namely, pyrazolinone or pyrazolinthione. For each of these individual rings, the NMR chemical shifts are generally very close to those of the respective ring type in (O,O)bimane or (S,S)bimane, i.e., within 0.10(7) ppm in the ^1^H NMR spectra and 1(2) ppm in the ^13^C NMR spectra, on average. This indicates that, as far as these nuclei are concerned, the two five-membered rings comprising the bimane core behave as two independent spin systems, each one being insensitive to thionation-induced electronic variations in the other. This differs markedly from the abovedescribed photophysical behavior of the bimanes, wherein thionation of only one of the pyrazolinone rings significantly alters the absorbance and fluorescence spectra of the entire bimane molecule.

The observed variations in chemical shifts upon thionation of the parent dioxobimanes can be rationalized on the basis of two opposing effects. As discussed above, the replacement of oxygen atoms by sulfur reduces the energy gap between the occupied and unoccupied frontier orbitals of the bimanes. This enhances the paramagnetic component of shielding, thereby pushing the NMR peaks downfield. At the same time, sulfur is much less electronegative than oxygen, and therefore the charge-separated resonance form typical of α,β-unsaturated carbonyls is expected to be far less dominant in the corresponding thiocarbonyls. Consequently, the partial positive charge at the β olefinic positions of the pyrazolinone rings drops significantly upon their thionation, resulting in the upfield-shifted peaks observed for the respective ^13^C nuclei of the pyrazolinthione rings.

### 2.5 X-ray Crystallography

The solid-state structures of thioxobimanes **3**–**6** were determined by single-crystal X-ray crystallography and are depicted in Table 3. Bimanes **3** and **4** cocrystallized with CDCl_3_ molecules in the *P*2_1_/*n* and *I*2 space groups, respectively. **5** and **6** crystallized in the *Pc* and *P*1 space groups, correspondingly, with no intercalated solvent molecules. The crystal structure of **5** exhibits significant positional disorder, with two coexisting components having opposite orientations. Only the major component, with 62% occupancy, is shown in Table 3 and is discussed here. The crystallographic data collected for bimanes **3**–**6** allow us to assess their structural attributes and compare them with those of the parent dioxobimanes **1** and **2**, as well as other previously reported dioxobimanes and related systems.

**Table 3:**
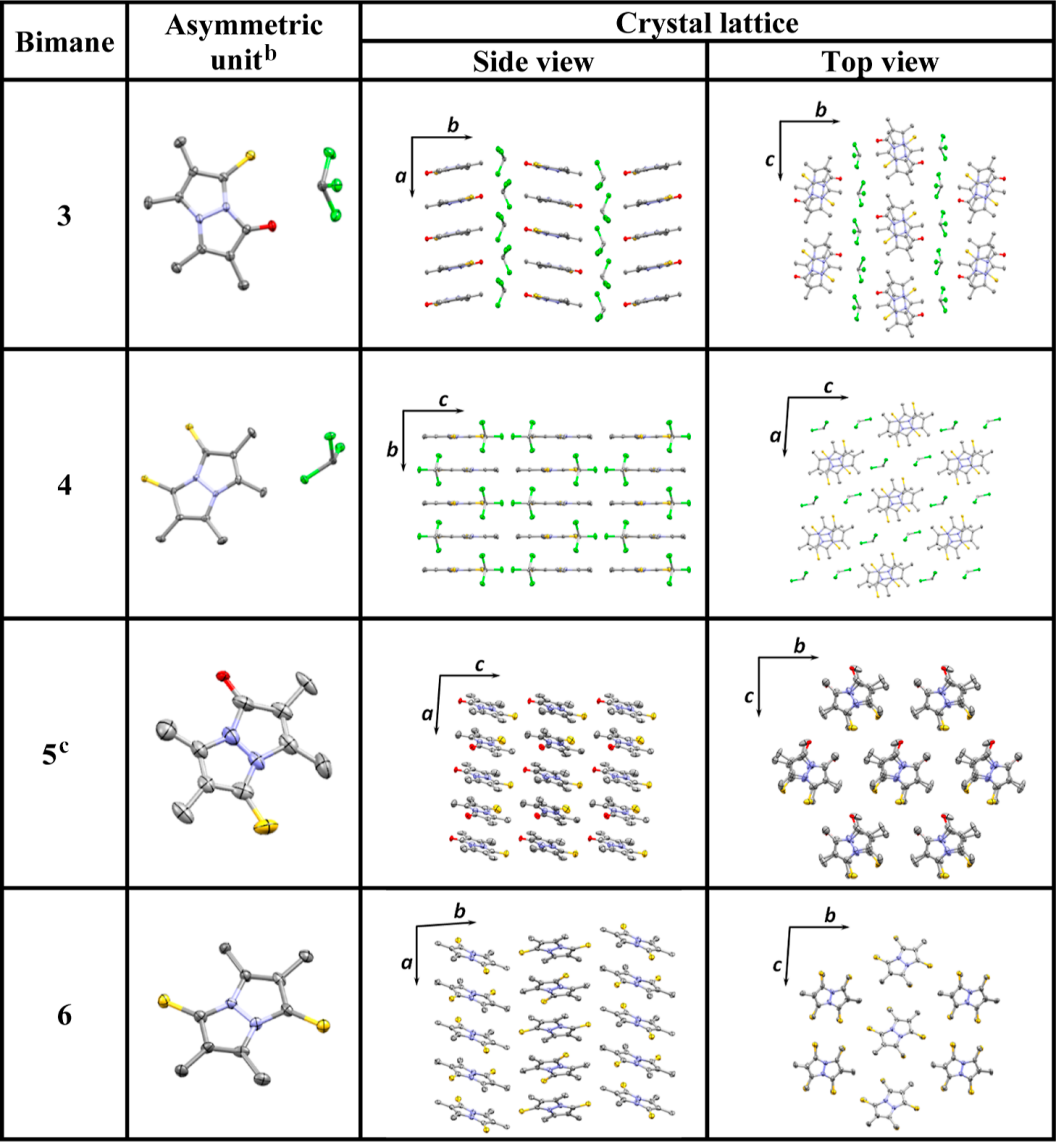
Crystal Structures of Thioxobimanes **3**–**6** Showing Their Asymmetric Units and Representative Sections of Their Crystal Lattices (ORTEP-Style with Ellipsoids at the 50% Probability Level; the Crystallographic Axes Perpendicular to the Viewing Direction Are Also Depicted)^a^

a The structures of **3** and **4** also include interstitial chloroform molecules. Atoms are color-coded as follows: C, gray; N, light blue; O, red; S, yellow; Cl, green. Hydrogen atoms have been omitted for clarity.

b **5** and **6** exhibit several bimane molecules per asymmetric unit, only one of which is shown here.

c The crystal lattice of **5** is disordered and comprises two components; only the predominant component (62% occupancy) is shown here.

The bond lengths and angles associated with the pyrazolinone and pyrazolinthione rings of **3**–**6** show little variation between these four thioxobimanes. Thus, when compared to the mean values across these thioxobimanes, their bond distances deviate by no more than 1.1(6)%, on average, and the bond angles by at most 1.5(8)%.^38^ Such close structural similarities are also found between the pyrazolinone rings of oxothioxobimanes **3** and **5** and their parent dioxobimanes **1** and **2**.^5,39,40^ The absence of literature examples of thioxobimanes precludes direct comparison of the pyrazolinthione metrics, but the Cambridge Structural Database (CSD; v5.43)^41^ does currently include four instances of relevant pyrazolinthione-containing compounds, which can be compared with thioxobimanes **3**–**6**. Here, too, the similarities are high, with the bond lengths of **3**–**6** deviating by at most 2(2)%, on average, from the mean values of the literature structures, and the bond angles varying by no more than 1.5(9)%.

The individual pyrazolinone and pyrazolinthione rings of the four thioxobimanes are expectedly planar, exhibiting very low average absolute torsion angles (τ̅) of 0.6(3) to 2.8(5)° for all ring bonds. The same is true of the intact bicyclic scaffolds of **4**–**6**, with τ̅ = 0.6(1)–2.2(5)° for their eight-membered rings, and essentially flat interplanar angles (φ) between the two five-membered rings of each thioxobimane, averaging 179.7(3)°. These features are all but identical to those of **1** and **2**, both of which are also planar.^5,39^ Bimane **3**, on the other hand, deviates somewhat from planarity, displaying slight butterfly bending of its bicyclic framework about the N–N bond, with φ = 172.6(1)° and τ̅ = 5(2)° for its eight-membered ring. The exact reason for this deformation of **3** is unclear, but it is a common characteristic of previously reported *syn*- and *anti*-dioxobimanes. Thus, out of the 12 *syn*- and 6 *anti*-dioxobimanes currently found in the CSD, which are simply bicyclic, half of the *syn*-bimanes and a third of the *anti*-bimanes are bent, whereas the rest are planar.^5,39,42−44^ The CSD contains 11 additional *syn*-dioxobimanes, but these exhibit a fused tricyclic framework, wherein the two pyrazolinone rings are mutually bridged through their β-olefinic positions by an aliphatic or heteroaliphatic chain. All of these tricyclic bimanes are bent about their N–N bonds, with most being strongly deformed.

The crystal structures of all four thioxobimanes display intermolecular stacking of these molecules, as can be seen in Table 3. This would be expected, considering their planarity, combined with the existence of highly conjugated π systems. In all cases, the thioxobimanes are stacked face-to-face, with a slippage of 1.3–1.9 Å between neighboring bimane molecules. The chalcogen atoms alternate in their positions along the stacked bimane columns, either on the same edge of the bicyclic bimane scaffold or on opposite edges. The stacking of thioxobimanes **3**, **4**, and **6** appears to involve π–π interactions, as evidenced by numerous short contacts between the rings of adjacent bimanes in a given column.^45^ In the case of **5**, no definitive short contacts were identified, but π–π interactions cannot be ruled out since its stacked molecules are in close proximity. Other notable intermolecular interactions are evident between *syn*-thioxobimanes **3** and **4** and their cocrystallized chloroform molecules. In both instances, there are short contacts between both chalcogen atoms of each thioxobimane and the carbon atom of chloroform, in the immediate vicinity of the carbon–hydrogen bond, indicating the existence of hydrogen-bonding. Moreover, in the crystal structure of **4**, each chloroform molecule appears to also be involved in chalcogen-halogen bonding with three nearby dithioxobimane molecules since a short contact exists between each of the chlorine atoms and a sulfur atom from a neighboring bimane.

### 2.6 *syn*-(Me,Me)Oxothioxobimane as a Ligand for Au(I)

**Scheme 2:**
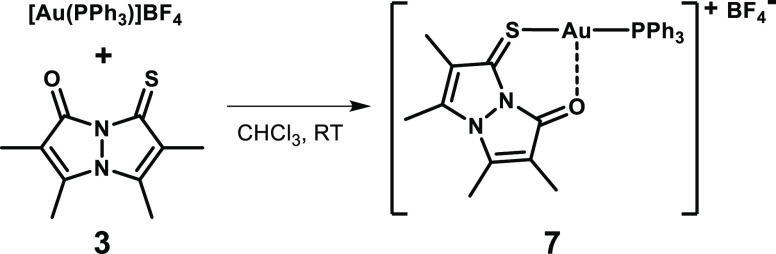
Synthesis of Au(I)-Thioxobimane Complex **7**

We have previously shown that bimane **1** can function as a weakly coordinating ligand for Pd(II),^15^ as well as for Li(I) and Na(I).^16,17^ We reasoned that replacing the carbonyl moieties of dioxobimanes by the softer thiocarbonyls will enhance coordination to many of the transition metals. As a proof-of-concept, we examined the room–temperature reaction of *syn*-(O,S)bimane **3** with the cationic gold(I) precursor [Au(PPh_3_)]BF_4_, obtained in situ by silver(I)-promoted chloride abstraction from Au(PPh_3_)Cl in chloroform. This reaction, which is outlined in Scheme 2, afforded the cationic Au(I)-oxothioxobimane complex **7**, which was isolated as orange crystals in 81% yield.

Complex **7** was characterized by both solution NMR spectroscopy (see Supporting Information) and X-ray crystallography. The ^31^P{^1^H} NMR spectrum of this complex in CDCl_3_ exhibits a singlet at 38.0 ppm, arising from the coordinated PPh_3_ ligand, and its ^19^F{^1^H} NMR spectrum displays a sharp singlet at −153.6 ppm, which is typical of noncoordinated BF_4_^–^. The ^1^H NMR spectrum of **7** displays four methyl resonances, namely, two peaks at 2.14 and 2.59 ppm, belonging to the pyrazolinthione ring, and two peaks at 1.86 and 2.52 ppm, belonging to the pyrazolinone ring. In each pair of CH_3_ peaks, the one representing the γ position is downfield of the one representing the β′ position, as was observed for the free thioxobimane **3**. However, the ^1^H NMR peaks arising from the Au(I) complex are shifted downfield by 0.20–0.25 ppm relative to the free thioxobimane, except for the pyrazolinone β′-CH_3_ resonance, which remains essentially unchanged. The effects of gold coordination to **3** are also clearly apparent in the ^13^C{^1^H} NMR spectrum, particularly vis-à-vis the thiocarbonyl carbon atom, with its associated peak being pushed strongly upfield, by over 15 ppm. By contrast, the carbonyl carbon resonance is only slightly affected, being shifted downfield by only 0.5 ppm. It is interesting to note that among the other ^13^C NMR peaks of **3**, only the olefinic resonances of the pyrazolinone ring vary by more than 1 ppm upon gold coordination. Thus, both of these peaks shift downfield, with the α position varying by 2.7 ppm and the β position by 6.0 ppm. Combined together, the ^1^H and ^13^C NMR data indicate that, in solution, the *syn*-oxothioxobimane strongly bonds to the Au(I) center through the sulfur atom of the pyrazolinthione ring but also interacts with the pyrazolinone ring. This coordination mode is indeed corroborated by the crystal structure of the gold complex.

**Figure 12:**
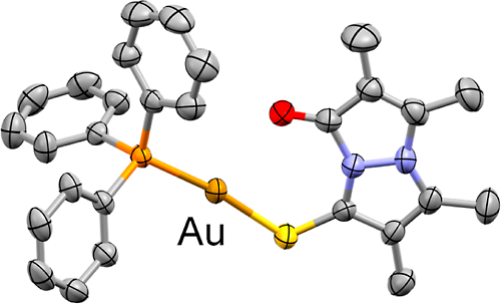
Crystal structure of the cationic fragment of complex **7** (ORTEP-style with ellipsoids at the 50% probability level). Atoms are color-coded as follows: C, gray; N, light blue; O, red; S, yellow; P, orange; Au, golden brown (labeled). Hydrogen atoms and the BF_4_^–^ counteranion have been omitted for clarity.

The crystal structure of complex **7** is shown in Figure 12. The complex, which crystallized in the *P*2_1_/*c* space group, exhibits *syn*-(O,S)bimane as a monodentate ligand, which is coordinated to the Au(I) center through the thiocarbonyl donor, in agreement with the aforementioned solution-phase NMR data. The bimane and PPh_3_ ligands define a slightly distorted linear coordination geometry, with ∠P–Au–S = 172.14(4)°, as would be expected of a d^10^ Au(I) center. The Au–S and Au–P bond lengths, at 2.308(1) and 2.262(1) Å, respectively, are virtually identical to the corresponding average bond lengths of the eight structurally similar gold(I)-PPh_3_-thiocarbonyl complexes currently found in the CSD [d̅(Au–S) = 2.31(2) Å, d̅(Au–P) = 2.268(7) Å]. Further examination of the structure of **7** reveals a notable short contact between the gold and oxygen atoms, with an interatomic distance of 2.751(3) Å, which is nearly 30% shorter than the sum of van der Waals radii of the two atoms.^45^ Although this Au–O distance is much longer than the Au–S bond, it does indicate the existence of a significant interaction between the carbonyl moiety of **3** and the Au(I) center. This, too, is consistent with the measured NMR data for complex **7**.

The coordinated *syn*-(O,S)bimane **3** slightly departs from planarity, in a manner similar to the free thioxobimane, with φ = 176.3(3)° and τ̅ = 3(1)° for its eight-membered ring. The bond lengths and angles associated with the bimane scaffold in complex **7** display very limited variation with respect to the free thioxobimane, i.e., its bond lengths deviate by only 1.6(9)%, on average, and its bond angles by a mere 0.9(6)%. Nevertheless, it is interesting to note that the largest differences in bond length, to the tune of ∼3%, are observed for the C=S bond of the pyrazolinthione ring, which elongates upon coordination, and the C=C bond of the pyrazolinone ring, which contracts. These same functionalities also display the largest variations in the solution-phase ^13^C NMR spectra, as described above.

### 2.7 Aromaticity of the Thioxobimanes

The fact that thioxobimanes **3**–**6**, as well as their lighter congeners **1** and **2**, exhibit highly conjugated ring structures prompted us to explore the aromaticity of these molecules. Aromaticity is typically discussed in terms of energetic, structural or magnetic criteria.^46−49^ The nucleus-independent chemical shift (NICS) is a standard magnetic measure of aromaticity,^46,47,50^ which is defined as the computed negative isotropic magnetic shielding at the center of a molecular ring structure, or at a given point along the axis perpendicular to the ring plane. The NICS parameter can be used to qualitatively assess the aromaticity of a given individual ring, or an entire polycyclic structure. It has many variants, of which we presently use NICS_zz_(1),^46,51^ which is the zz component of NICS at a distance of 1.0 Å from the ring (*xy*) plane, providing a measure of π aromaticity. In general terms, a highly negative (shielded) NICS indicates aromaticity, whereas a large positive (deshielded) NICS implies antiaromaticity, and a small negative or positive NICS is associated with a non-aromatic character.^46,52^ For bimanes **1, 2, 4**, and **6**, each of which consists of two identical five-membered rings—either pyrazolinone or pyrazolinthione—NICS_zz_(1) was computed above the geometric center of only one ring per molecule. In the case of bimanes **3** and **5**, each of which comprises two different rings, NICS_zz_(1) indices were calculated for both rings of each molecule.

**Figure 13:**
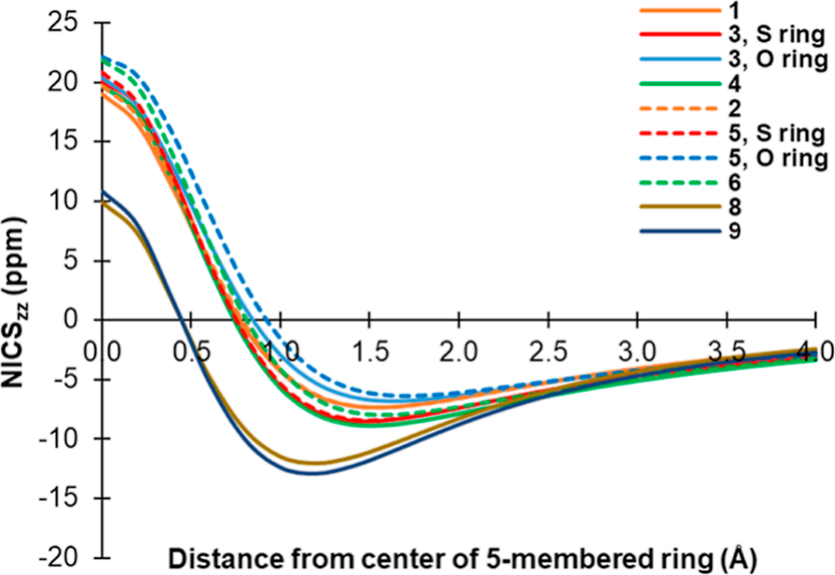
NICS_zz_ values for *syn*-bimanes **1**, **3**, and **4** (solid lines), *anti*-bimanes **2**, **5**, and **6** (dotted lines), and model compounds **8** and **9**, as a function of distance from the center of their constituent five-membered rings along the *z*-axis (at 0.2 Å increments). O ring = pyrazolinone; S ring = pyrazolinthione.

For the homochalcogenide *syn*-bimanes **1** and **4**, NICS_zz_(1) amounts to −4.3 and −5.8 ppm, respectively, whereas for the mixed-chalcogenide *syn*-bimane **3**, it is −3.0 ppm for the pyrazolinone ring and −5.6 ppm for the pyrazolinthione ring. Similar results were obtained for the respective *anti*-bimanes, with **2** and **6** exhibiting nearly identical NICS_zz_(1) values of −4.2 and −4.1 ppm, respectively, and **5** showing values of −1.6 and −5.3 ppm for the pyrazolinone and pyrazolinthione rings, respectively. These small negative values suggest that all six bimane molecules are either non-aromatic or only weakly so.^52^ It should be noted that previous computational studies based on NICS(1), computed on the concave face of the bimane molecules, also yielded small negative values of −3.6 and −3.1 ppm for **1** and **2**, respectively, in good agreement with our results.^53^ To further elucidate the nature of aromaticity in these bimane molecules, we computed NICS_zz_ as a function of distance along the *z*-axis, from the center of their constituent five-membered rings up to a distance of 4 Å, at 0.2 Å increments. The resulting plots, depicted in Figure 13, behave similarly for all six bimanes. Thus, at the very center of the ring, NICS_zz_(0) exhibits large positive values, ranging from 18.9 to 22.1 ppm, which account for a combination of σ and π aromaticity. Upon moving away from the ring plane, the effect of σ electrons wanes whereas that of the π electrons becomes dominant. Consequently, NICS_zz_ decreases as a function of distance, reaching a minimal negative value around 1.5 Å, ranging from −6.8 to −8.9 ppm. Moving further away, the NICS_zz_ indices of all six bimanes slightly increase, roughly converging to about −5 ppm at 3.0 Å and eventually reaching 0 ppm at infinite separation. This type of fluctuation of the NICS_zz_ profile, from a positive value to a negative minimum and then convergence to zero at large distances, does not fit the behavior of distinctly aromatic or antiaromatic molecules^46^ but is typical of molecules showing low or no aromaticity.^52,54^

**Figure 14:**
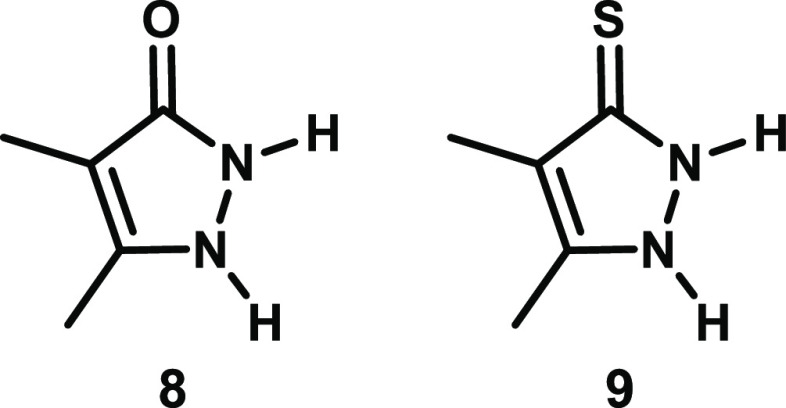
Structures of pyrazolinone **8** and pyrazolinthione **9**, used as computational models to assess the aromaticity of the individual five-membered rings comprising the bicyclic bimane scaffolds.

For comparative purposes, we also computed the NICS_zz_ profiles of model pyrazolinone **8** and pyrazolinthione **9** (Figure 14), in order to investigate the aromaticity of individual five-membered rings that are structurally similar to those comprising the bicyclic bimane scaffolds. As can be seen in Figure 13, both **8** and **9** exhibit profiles that are similar to those of the bimanes but begin at lower NICS_zz_(0) values and display deeper minima, with more negative NICS_zz_ values of −12.9 and −12.0 ppm, respectively, at a distance of only 1.2 Å. This indicates that these monocyclic structures have higher aromatic character than the bicyclic bimanes themselves, which appear to be only weakly- or non-aromatic. Thus, conceptually fusing two pyrazolinone or pyrazolinthione rings, or a combination of both, into an intact bimane molecule results in diminished aromaticity.

## 3 Conclusions

In the present report, we described the synthesis and characterization of thioxobimanes, the first heavy-chalcogenide variants of bimanes, a family of *N*-heterobicyclic compounds introduced by Kosower and co-workers over four decades ago. The new sulfur-containing bimanes, **3**–**6**, were prepared in fair-to-good yields through thionation of *syn*- and *anti*-(Me,Me)dioxobimane (**1** and **2**), using Lawesson’s reagent and P_4_S_10_. They were then subjected to extensive analysis, including UV–vis, IR, Raman, and NMR spectroscopies, as well as X-ray crystallography. Our results show that introducing sulfur atoms into the bimane core does not lead to appreciable structural changes but does significantly modulate the absorption maxima of all bimanes and effectively eliminates the original *syn*-bimane fluorescence in the UV–vis region. As mentioned above, this could be utilized for the development of thioxobimane-based turn-on fluorescent chemosensors. The vibrational and NMR spectra of the various bimanes also exhibit noticeable differences, in line with the different nature of the C=S *vs* C=O bond and the inherent variations in molecular symmetry. We have also conducted a computational study of bimane aromaticity, showing that both the dioxo- and thioxobimanes are only weakly- or non-aromatic, despite the apparent existence of extensive π conjugation within the bimane ring systems. Lastly, we synthesized and characterized a gold(I) complex of thioxobimane **3**, thereby demonstrating the ability of these new bimane derivatives to serve as ligands for transition metals.

## 4 Experimental Section

### 4.1 General Procedures

All solvents and commercially available reagents were of reagent grade or better and were used without further purification. Heating of reaction mixtures, when required, was done by immersing the reaction vessel in a silicone oil heating bath set at the appropriate temperature. Bimanes **1** and **2** were synthesized according to published procedures.^2,6^ NMR spectra were processed with TopSpin 3.5 (Bruker Corp.)^55^ and plotted using MestReNova (v14.3.1).^56^ Previously published crystal structures were retrieved from the CSD (v5.43, September 2022)^41,57^ using ConQuest 2022.2.0,^58^ and new crystal structures were drawn using Mercury 4.3.1.^59^

### 4.2 Analysis

^1^H, ^13^C{^1^H}, ^15^N{^1^H}, ^19^F{^1^H}, and ^31^P{^1^H} NMR spectra were recorded using a Bruker Avance-III 400 MHz spectrometer, equipped with a 5 mm BBFO SmartProbe. All measurements were done at 25 °C. ^1^H and ^13^C NMR chemical shifts are reported in ppm relative to tetramethylsilane and referenced to the residual protium signal of the deuterated solvent and the ^13^C signals of this solvent, respectively. Assignment of the ^1^H and ^13^C NMR signals was confirmed by HMBC and HMQC experiments (see Supporting Information), which were complemented by a 1,1-ADEQUATE experiment in the case of bimane **6**. ^15^N NMR chemical shifts are reported in ppm relative to CH_3_NO_2_ and referenced to an external sample of 90% CH_3_NO_2_ in CDCl_3_ (δ = 0 ppm). All ^15^N NMR data were obtained from ^15^N–^1^H HMBC experiments (see Supporting Information). ^19^F NMR chemical shifts are reported in ppm relative to CFCl_3_ and referenced to an external solution of 0.05% α,α,α-trifluorotoluene in CDCl_3_ (δ = −62.7 ppm). ^31^P NMR chemical shifts are reported in ppm relative to H_3_PO_4_ and referenced to an external sample of 85% aqueous H_3_PO_4_ (δ = 0 ppm). Abbreviations used in the description of NMR data are as follows: s, singlet; q, quartet; m, multiplet; br, broad.

UV–vis absorbance spectra were measured with a Varian Cary 100 Bio UV–Visible Spectrophotometer. Fluorescence spectra in the UV–vis region were recorded on a Varian Cary Eclipse Fluorescence Spectrophotometer. IR spectra were recorded in the ATR mode using a Bruker Vertex 70 FT-IR spectrometer, fitted with a PLATINUM Diamond ATR accessory. Raman spectra were acquired using a Horiba LabRAM Evolution HR spectrometer equipped with a high-resolution 800 mm focal length spectrograph. High-resolution mass spectrometry (HRMS) data for complex **7** were collected using a Xevo G2-XS-QTof device. HRMS data for bimanes **3**–**6** were measured at the Mass Spectrometry Lab, Schulich Faculty of Chemistry, Technion - Israel Institute of Technology, using a Bruker Maxis Impact quadrupole time-of-flight (QTOF) HRMS instrument.

Crystals of thioxobimanes **3**–**6** that are suitable for X-ray diffraction were grown under various conditions, as follows: **3** was crystallized from CDCl_3_ at −20 °C, affording orange needles; **4** and **6** were crystallized at −20 °C from their respective CDCl_3_ solutions overlaid with diethyl ether, forming pink and red needles, respectively; and **5** crystallized as yellow needles from an acetonitrile solution exposed to diethyl ether vapors at room temperature. The gold complex **7** was crystallized at room temperature from a CHCl_3_ solution overlaid with diethyl ether, affording orange plates.

X-ray diffraction data for crystals of thioxobimanes **3**–**6** were collected at 100K on a Rigaku Xtalab PRO dual-source diffractometer equipped with a Dectris Pilatus 200K detector and microfocus, using CuKα radiation (λ = 1.54184 Å). The data were processed with CrysAlis^PRO^. X-ray diffraction data for a crystal of complex **7** were measured at room temperature on a Bruker APEX-II Kappa CCD diffractometer, using MoKα (λ = 0.71073 Å) radiation. These data were processed with SAINT. Crystal structures were solved by direct methods using SHELXT,^60^ and the data were refined by full-matrix least-squares techniques, based on F^2^, using SHELXL^61^ and OLEX2.^62^ All non-hydrogen atoms were further refined with anisotropic displacement coefficients, whereas hydrogen atoms were assigned isotropic displacement coefficients, and their coordinates were allowed to ride on their respective carbon atoms. Crystallographic data and refinement parameters are compiled in Table S1 (see Supporting Information). The crystallographic coordinates for the structures reported in this study have been deposited at the Cambridge Crystallographic Data Centre (CCDC) under deposition numbers 2091690–2091694. These data can be obtained free of charge at https://www.ccdc.cam.ac.uk/data_request/cif. Crystal structures were analyzed using Mercury 4.3.1^59^ and Platon 10421 (for Microsoft Windows).^63,64^

### 4.3 Synthesis

#### 4.3.1 Synthesis of *syn*-(Me,Me)Oxothioxobimane (**3**)

A mixture of **1** (576 mg, 3.00 mmol) and Lawesson’s reagent (724 mg, 1.79 mmol) in 50 mL of toluene was refluxed for 1 h under an N_2_ atmosphere. The reaction mixture was then subjected to flash column chromatography over silica, under air, using a 3:7 (v/v) petroleum ether/ethyl acetate mixture as the eluent. The relevant fractions were collected and combined, and the solvent was evaporated under reduced pressure. The remaining residue was then dissolved in CHCl_3_, and the solution was stored at −20 °C to induce product crystallization. This afforded 446 mg (2.14 mmol, 71% yield) of the product as orange needle-like crystals.

^1^H NMR (400 MHz, CDCl_3_): 2.34 (poorly resolved q, 3H, C*H*_3_, γ position of the pyrazolinthione ring), 2.32 (q, ^5^*J*_HH_ = 1.0 Hz, 3H, C*H*_3_, γ position of the pyrazolinone ring), 1.93 (poorly resolved q, 3H, C*H*_3_, β′ position of the pyrazolinthione ring), and 1.85 (q, ^5^*J*_HH_ = 1.0 Hz, 3H, C*H*_3_, β′ position of the pyrazolinone ring). ^13^C{^1^H} NMR (101 MHz, CDCl_3_): 177.5 (s, *C*=S), 159.4 (s, *C*=O), 141.8 (s, *C*=C, β position of the pyrazolinone ring), 141.2 (s, *C*=C, β position of the pyrazolinthione ring), 124.7 (s, *C*=C, α position of the pyrazolinthione ring), 112.1 (s, *C*=C, α position of the pyrazolinone ring), 11.4 (s, *C*H_3_, γ position of the pyrazolinthione ring), 11.4 (s, *C*H_3_, γ position of the pyrazolinone ring), 8.3 (s, *C*H_3_, β′ position of the pyrazolinthione ring), and 6.8 (s, *C*H_3_, β′ position of the pyrazolinone ring). ^15^N{^1^H} NMR (41 MHz, CDCl_3_): −196 (β position). ESI-HRMS (CH_3_CN): M + H^+^, *m*/*z* 209.0684; calcd for C_10_H_12_N_2_OS + H^+^, 209.0749.

#### 4.3.2 Synthesis of *syn*-(Me,Me)Dithioxobimane (4)

A mixture of **1** (1.00 g, 5.20 mmol) and P_4_S_10_ (2.51 g, 5.65 mmol) in 110 mL of benzene was refluxed for 1 h under an N_2_ atmosphere. The reaction mixture was then subjected to gravity column chromatography over silica, under air, using CH_2_Cl_2_ as the eluent. The relevant fractions were collected and combined, and the solvent was evaporated under reduced pressure. The remaining residue was then dissolved in CHCl_3_, and the solution was overlaid with diethyl ether and stored at −20 °C to induce product crystallization. This procedure afforded 785 mg (3.50 mmol, 67% yield) of the product as deep-purple hair-like crystals.

^1^H NMR (400 MHz, CDCl_3_): 2.40 (poorly resolved q, 6H, 2C*H*_3_, γ position), 2.05 (poorly resolved q, 6H, 2C*H*_3_, β′ position). ^13^C{1H} NMR (101 MHz, CDCl_3_): 176.0 (s, *C*=S), 136.5 (s, *C*=C, β position), 124.5 (s, *C*=C, α position), 11.0 (s, 2*C*H_3_, γ position), and 8.7 (s, 2*C*H_3_, β′ position). ^15^N{1H} NMR (41 MHz, CDCl_3_): −180.6 (β position). ESI-HRMS (CH_3_CN): M + H^+^, *m*/*z* 225.0456; calcd for C_10_H_12_N2S_2_ + H^+^, 225.0520.

#### 4.3.3 Synthesis of *anti*-(Me,Me)Oxothioxobimane (**5**)

A mixture of **2** (100 mg, 0.520 mmol) and Lawesson’s reagent (168 mg, 0.415 mmol) in 15 mL of benzene was refluxed for 6 h under an N_2_ atmosphere. The reaction mixture was then subjected to gravity column chromatography over silica, under air, using a 1:9 (v/v) petroleum ether/ethyl acetate mixture as the eluent. The relevant fractions were collected and combined, and the solvent was evaporated under reduced pressure. The remaining residue was then dissolved in acetone, and product crystallization was induced by exposing the resulting solution to diethyl ether vapors for several days at room temperature. This afforded 46.0 mg (0.221 mmol, 43% yield) of the product as yellow needle-like crystals.

^1^H NMR (400 MHz, CDCl_3_): 2.65 (poorly resolved q, 3H, C*H*_3_, γ position of the pyrazolinone ring), 2.45 (poorly resolved q, 3H, C*H*_3_, γ position of the pyrazolinthione ring), 1.95 (poorly resolved q, 3H, C*H*_3_, β′ position of the pyrazolinthione ring), and 1.86 (poorly resolved q, 3H, C*H*_3_, β′ position of the pyrazolinone ring). ^13^C{^1^H} NMR (101 MHz, CDCl_3_): 178.2 (s, *C*=S), 158.9 (s, *C*=O), 146.2 (s, *C*=C, β position of the pyrazolinone ring), 140.8 (s, *C*=C, β position of the pyrazolinthione ring), 125.0 (s, *C*=C, α position of thepyrazolinthione ring), 113.1 (s, *C*=C, α position of the pyrazolinone ring), 12.8 (s, *C*H_3_, γ position of the pyrazolinone ring), 10.1 (s, *C*H_3_, γ position of the pyrazolinthione ring), 8.3 (s, *C*H_3_, β′ position of the pyrazolinthione ring), and 6.4 (s, *C*H_3_, β′ position of the pyrazolinone ring). ^15^N{^1^H} NMR (41 MHz, CDCl_3_): −180.8 (α to C=O), −176.9 (α to C=S). ESI-HRMS (CH_3_CN): M + H^+^, *m*/*z* 209.0653; calcd for C_10_H_12_N_2_OS + H^+^, 209.0749.

#### 4.3.4 Synthesis of *anti*-(Me,Me)Dithioxobimane (**6**)

A mixture of **2** (138 mg, 0.718 mmol) and P_4_S_10_ (477 mg, 1.07 mmol) in 20 mL of toluene was refluxed for 0.5 h under an N_2_ atmosphere. The reaction mixture was then subjected to flash column chromatography over silica, under air, using a 2:3 (v/v) petroleum ether/ethyl acetate mixture as the eluent. The relevant fractions were collected and combined, and the solvent was evaporated under reduced pressure. The remaining residue was then dissolved in CHCl_3_, and product crystallization was induced by exposing the resulting solution to diethyl ether vapors for several days at −20 °C. This afforded 66.0 mg (0.294 mmol, 41% yield) of the product as red hair-like crystals.

^1^H NMR (400 MHz, CDCl_3_): 2.63 (poorly resolved q, 6H, 2C*H*_3_, γ position), 1.99 (poorly resolved q, 6H, 2C*H*_3_, β′ position). ^13^C{^1^H} NMR (101 MHz, CDCl_3_): 175.7 (s, *C*=S), 141.5 (s, *C*=C, β position), 125.2 (s, *C*=C, α position), 12.4 (s, 2*C*H_3_, γ position), and 8.3 (s, 2*C*H_3_, β′ position). ^15^N{^1^H} NMR (41 MHz, CDCl_3_): −162.9. ESI-HRMS (CH_3_CN): M + H^+^, *m*/*z* 225.0476; calcd for C_10_H_12_N_2_S_2_ + H^+^, 225.0520.

#### 4.3.5 Synthesis of *trans*-{Au[*syn*-(Me,Me)Oxothioxobimane-κ*S*](PPh_3_)}BF_4_ (**7**)

Au(PPh_3_)Cl (90 mg, 0.182 mmol) and AgBF_4_ (35.4 mg, 0.182 mmol) were mixed with 6.0 mL of CHCl_3_, and the resulting suspension was stirred at room temperature, in the dark, for 1.5 h. The reaction mixture was then filtered through a cotton pad to remove the AgCl precipitate and afford a clear solution. Solid bimane **3** (38.0 mg, 0.182 mmol) was added to this solution, and the resulting orange-brown solution was stirred at room temperature for 1 h. The solvent was then evaporated under reduced pressure, and the remaining residue was redissolved in CHCl_3_. The product was crystallized by overlaying the CHCl_3_ solution with diethyl ether at room temperature. This afforded 111 mg (0.147 mmol, 81% yield) of the product as orange plate-shaped crystals.

^1^H NMR (400 MHz, CDCl_3_): 7.54 (m, 3H, Ar–*H*, PPh_3_), 7.53 (m, 6H, Ar–*H*, PPh_3_), 7.52 (m, 6H, Ar–*H*, PPh_3_), 2.59 (br s, 3H, C*H*_3_, γ position of the pyrazolinthione ring), 2.52 (q, ^5^*J*_HH_ = 1.0 Hz, 3H, C*H*_3_, γ position of the pyrazolinone ring), 2.14 (br s, 3H, C*H*_3_, β′ position of the pyrazolinthione ring), and 1.86 (q, ^5^*J*_HH_ = 1.0 Hz, 3H, C*H*_3_, β′ position of the pyrazolinone ring). ^13^C{^1^H} NMR (101 MHz, CDCl_3_): 162.4 (s, *C*=S), 159.9 (s, *C*=O), 147.2 (s, *C*=C, β position of the pyrazolinone ring), 141.1 (s, *C*=C, β position of the pyrazolinthione ring), 134.0 (d, *J*_PC_ = 13.7 Hz, *C*Ar–H), 132.2 (d, *J*_PC_ = 2.4 Hz, *C*Ar–H), 129.4 (d, *J*_PC_ = 11.8 Hz, *C*Ar–H), 128.2 (d, ^1^*J*_PC_ = 60.1 Hz, *C*Ar–P), 124.9 (s, *C*=C, α position of the pyrazolinthione ring), 114.8 (s, *C*=C, α position of the pyrazolinone ring), 11.7 (s, *C*H_3_, γ position of the pyrazolinthione ring), 11.5 (s, *C*H_3_, γ position of the pyrazolinone ring), 9.0 (s, *C*H_3_, β′ position of the pyrazolinthione ring), and 6.9 (s, *C*H_3_, β′ position of the pyrazolinone ring). ^19^F{^1^H} NMR (376 MHz, CDCl_3_): −153.6 (s, noncoordinated B*F*_4_^–^). ^31^P{^1^H} NMR (162 MHz, CDCl_3_): 38.0 (s, Au–*P*Ph_3_). ESI-HRMS (CH_3_CN): M^+^, *m*/*z* 667.1278; calcd for [C_28_H_27_AuN_2_OPS]^+^, 667.1247.

### 4.4 Computational Methods

Molecular geometries and harmonic vibrational frequencies were computed in the gas phase using DFT at the B3LYP^65^/6-311+G(d,p) level, employing the Gaussian 16 software suite.^66^ All of the calculated vibrational frequencies were scaled by a factor of 0.96979. For assessment of aromaticity, absolute NMR shielding values were calculated using the gauge-independent atomic orbital method^67^ at the same level of theory. These data were then used for computing NICS^46^ values, which are commonly employed magnetic criteria for aromaticity, with particular focus on their zz components (NICS_zz_). These calculations were carried out by placing a NICS (“BQ”) probe on the convex side of a given bimane molecule, above the geometric center of each five-membered ring of this molecule, at a distance of 0.0–4.0 Å from the ring (*xy*) plane along a perpendicular line (*z*-axis) at 0.2 Å increments.

## Data Availability

The data underlying this report are available in the published article and its Supporting Information.

## Supporting Information Available

Crystallographic data and refinement parameters, experimental NMR spectra (^13^C{^1^H}, ^13^C–^1^H HMBC and HMQC, ^15^N–^1^H HMBC), preliminary results regarding the effect of oxidants on the fluorescence of solutions containing thioxobimane **4**, data tables for the calculated bimane vibrational frequencies, and Cartesian coordinates of the optimized bimane geometries (PDF)
