# Supplementary material for: Thioxobimanes
Source: J Org Chem. 2023 Sep 15;88(19):13475–89. doi: 10.1021/acs.joc.3c00873 (PMC10563133; doi:10.1021/acs.joc.3c00873)
Supplement: Supplementary file 1 — jo3c00873_si_001.pdf [file jo3c00873_si_001.pdf]

Supporting Information

Thioxobimanes

Partha Jyoti Das,[1] Ankana Roy,[1] Ashim Nandi,[2] Ishita Neogi,[1]

Yael Diskin-Posner,[3] Vered Marks,[1] Iddo Pinkas,[3] Sara Amer,[1]

Sebastian Kozuch,[2] Michael Firer,[4] Michael Montag,[*,1] Flavio Grynszpan,[*,1]

[1] Department of Chemical Sciences, Ariel University, Ariel 40700, Israel, Fax: (+972)3-906-6634
E-mails: michael.montag@weizmann.ac.il; flaviog@ariel.ac.il

[2] Department of Chemistry, Ben-Gurion University, Beer Sheva 841051, Israel

[3] Department of Chemical Research Support, Weizmann Institute of Science, Rehovot 76100, Israel

[4] Department of Chemical Engineering and Biotechnology, Ariel University, Ariel 40700, Israel

Table of contents

Crystallographic data	S1
NMR spectra	S2-S12
Effect of oxidants on the fluorescence of solutions containing thioxobimane **4**	S13
Computed vibrational frequencies	S14
Cartesian coordinates of computed geometries	S15-S18

Table S1. Crystallographic data for thioxobimanes 3-6 and complex 7.

Species	3	4	5	6	7
CCDC number	2091694	2091690	2091692	2019693	2091691
Formula	$C_{10}H_{12}N_2OS•CHCl_3$	$C_{10}H_{12}N_2S_2•CHCl_3$	$C_{10}H_{12}N_2OS$	$C_{10}H_{12}N_2S_2$	$C_{28}H_{27}AuN_2OPS•BF_4$
Formula weight (g mol^{-1})	327.64	343.70	208.28	224.34	754.32
Crystal system	Monoclinic	Monoclinic	Monoclinic	Triclinic	Monoclinic
Space group	$P2_1/n$	I_2	Pc	$P\bar{1}$	$P2_1/c$
Crystal size (µm)	161×92×67	152×82×52	159×54×49	95×31×21	100×10×10
Crystal color and shape	Orange prism	Red prism	Orange prism	Red needle	Orange plate
Temperature (K)	100	100	100	100	296
Wavelength (Å)	1.54184	1.54184	1.54184	1.54184	0.71073
a (Å)	6.9584(1)	11.47754(11)	7.39370(5)	7,7681(2)	14.3670(4)
b (Å)	20.9830(3)	6.87231(6)	9.17116(6)	16.1521(3)	12.5156(4)
c (Å)	9.8136(1)	19.47681(17)	14.86069(11)	16.8030(4)	16.8968(5)
α (°)	90	90	90	94.878(2)	90
β (°)	93.040(1)	97.7684(8)	93.6493(6)	94.408(2)	110.2760(10)
γ (°)	90	90	90	94.427(2)	90
Volume (Å^3)	1430.85(3)	1522.18(2)	1005.642(12)	2086.99(8)	2849.97(15)
Z	4	4	4	8	4
$\rho_{calcd,}$(g cm^{-3})	1.521	1.500	1.376	1.428	1.758
µ (mm^{-1})	7.083	7.884	2.596	4.291	5.343
Number of reflections (unique)	24101 (2934)	6736 (2496)	16704 (3184)	58992 (8210)	34638 (6540)
R_{int}	0.0423	0.0251	0.0282	0.0543	0.0397
Completeness to θ (%)	99.9	99.8	98.0	99.5	99.7
Data / restraints / parameters	2934 / 0 / 167	2496 / 1 / 168	3184 / 8 / 347	8210 / 0 / 521	6540 / 0 / 356
Goodness-of-fit on F^2	1.091	1.087	1.122	1.083	0.995
Final R_1 and wR_2 indices [$I > 2\sigma(I)$]	0.0443, 0.1135	0.0288, 0.0803	0.0454, 0.1310	0.0534, 0.1514	0.0310, 0.0691
R_1 and wR_2 indices (all data)	0.0465, 0.1148	0.0293, 0.0808	0.0478, 0.1357	0.0908, 0.1769	0.0512, 0.0770
Largest difference: peak, hole (e Å^{-3})	0.774, -0.298	0.235, -0.416	0.196, -0.241	0.737, -0.347	1.353, -0.502

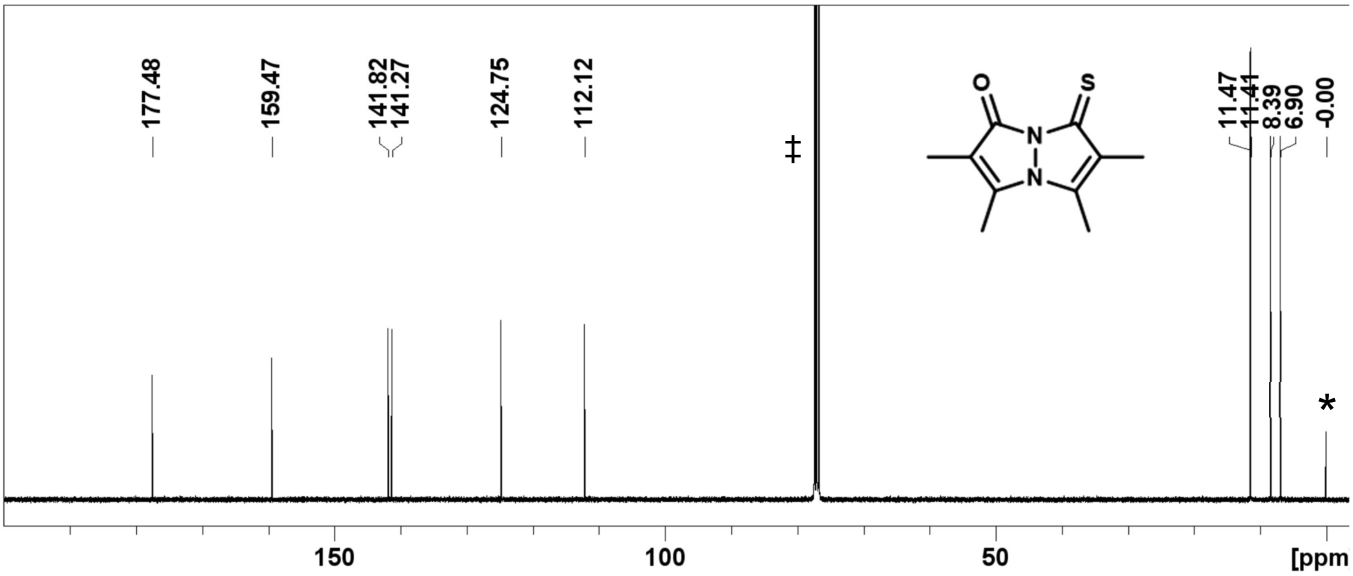

Figure S1. $^{13}C\{^1H\}$ NMR spectrum (101 MHz) of *syn*-bimane **3** in $CDCl_3$ (*, tetramethylsilane; ‡, solvent peak).

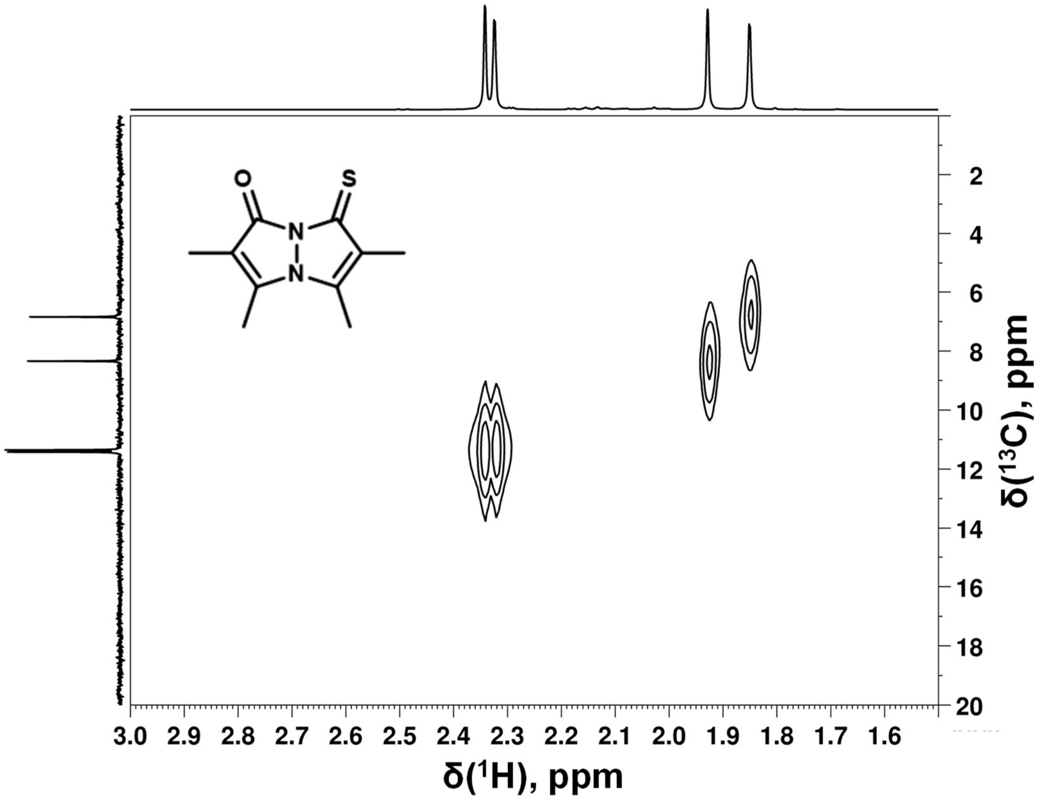

Figure S2. ^{13}C-^1H HMQC spectrum (101 MHz / 400 MHz) of *syn*-bimane **3** in $CDCl_3$.

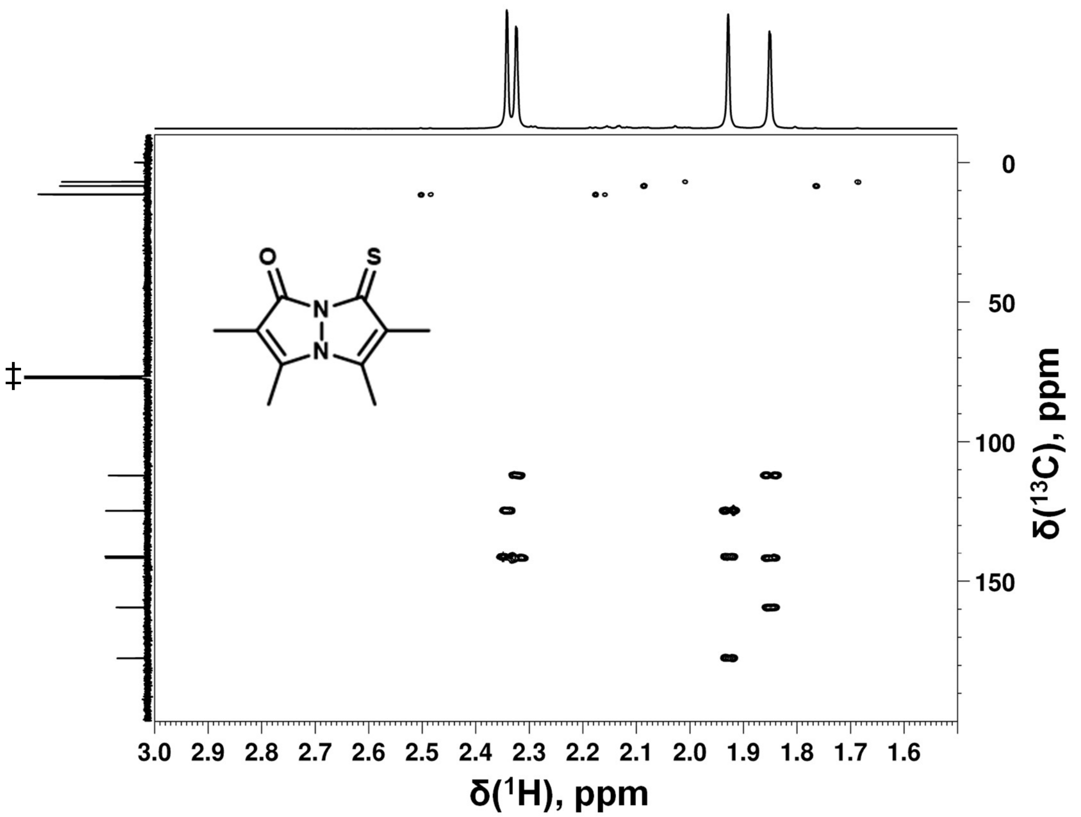

Figure S3. ^{13}C-^{1}H HMBC spectrum (101 MHz / 400 MHz) of *syn*-bimane **3** in $CDCl_3$ (‡, solvent peak).

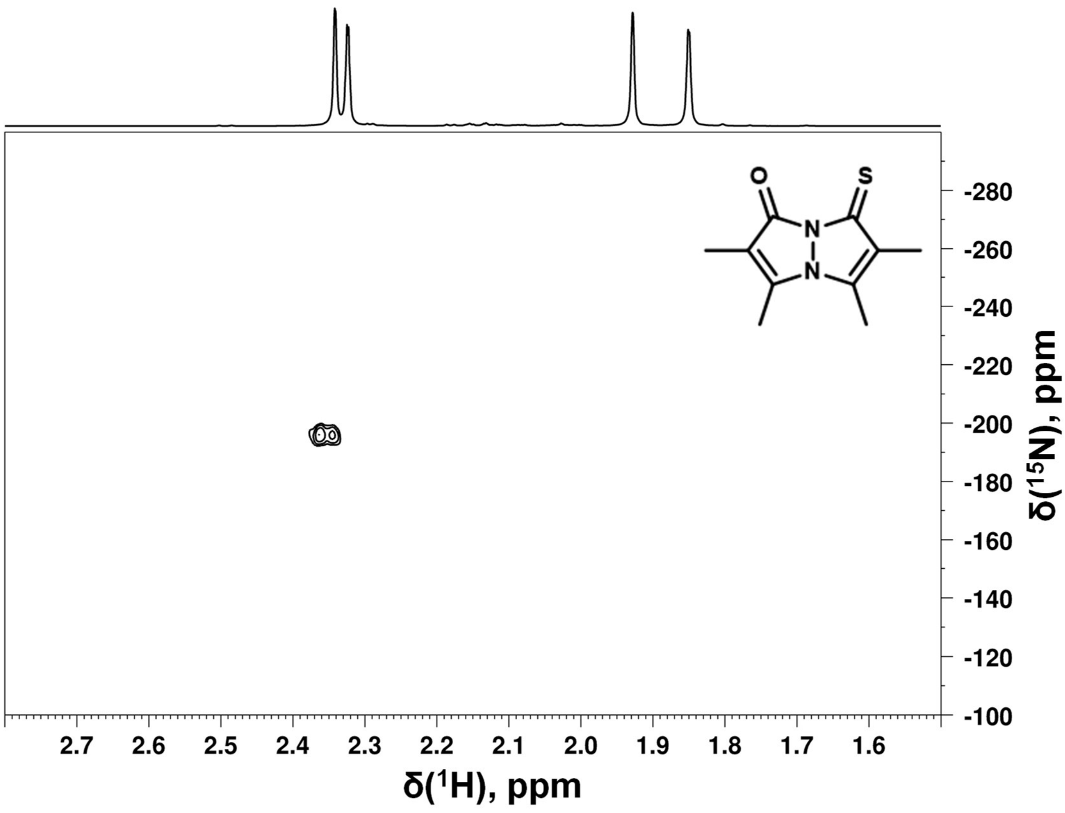

Figure S4. ^{15}N-^{1}H HMBC spectrum (41 MHz / 400 MHz) of *syn*-bimane **3** in $CDCl_3$.

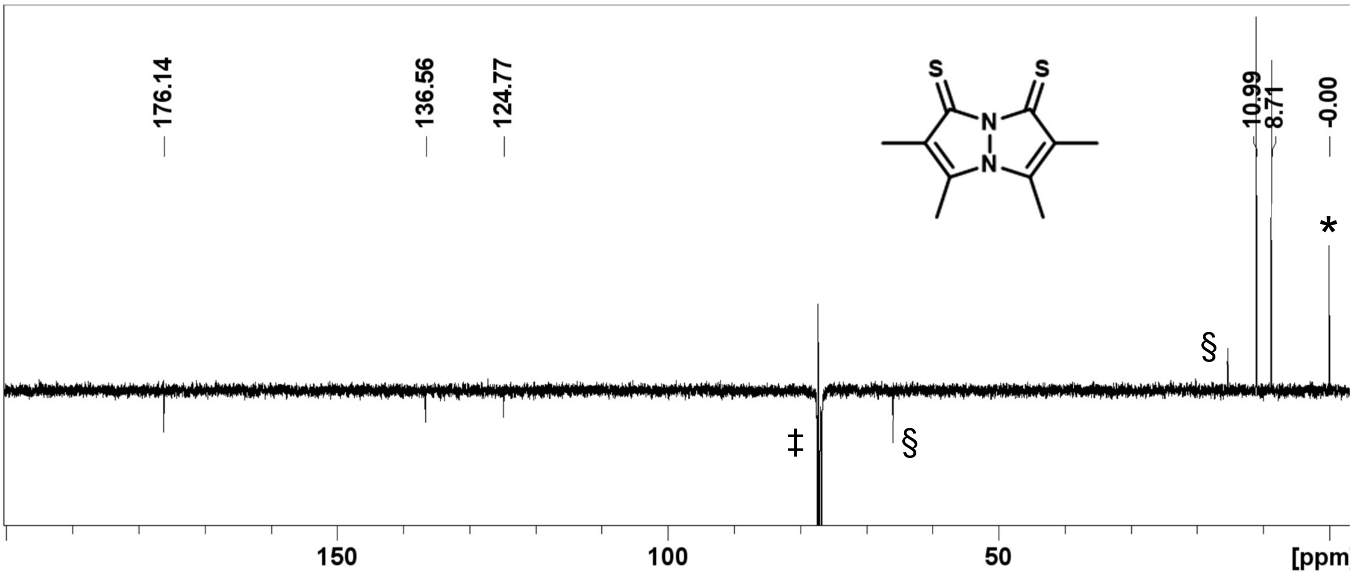

Figure S5. $^{13}C\{^1H\}$ DEPTQ NMR spectrum (101 MHz) of *syn*-bimane **4** in $CDCl_3$ (*, tetramethylsilane; ‡, solvent peak; §, residual diethyl ether).

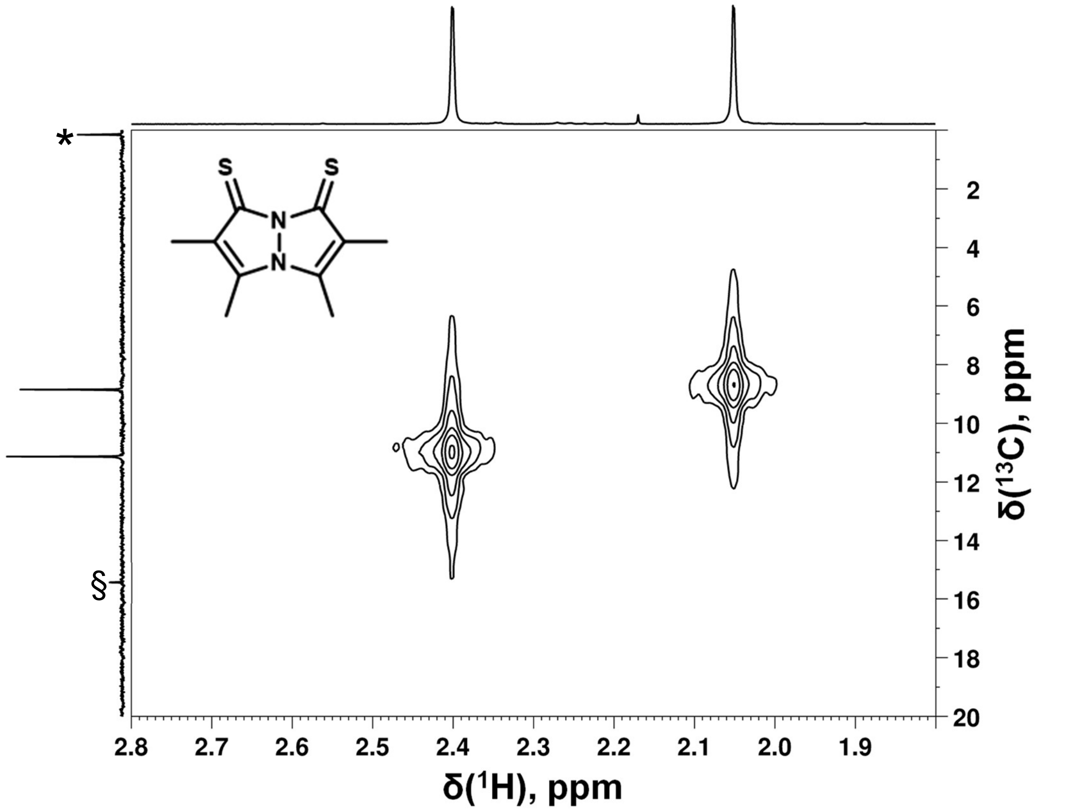

Figure S6. ^{13}C-^1H HMQC spectrum (101 MHz / 400 MHz) of *syn*-bimane **4** in $CDCl_3$ (*, tetramethylsilane; §, residual diethyl ether).

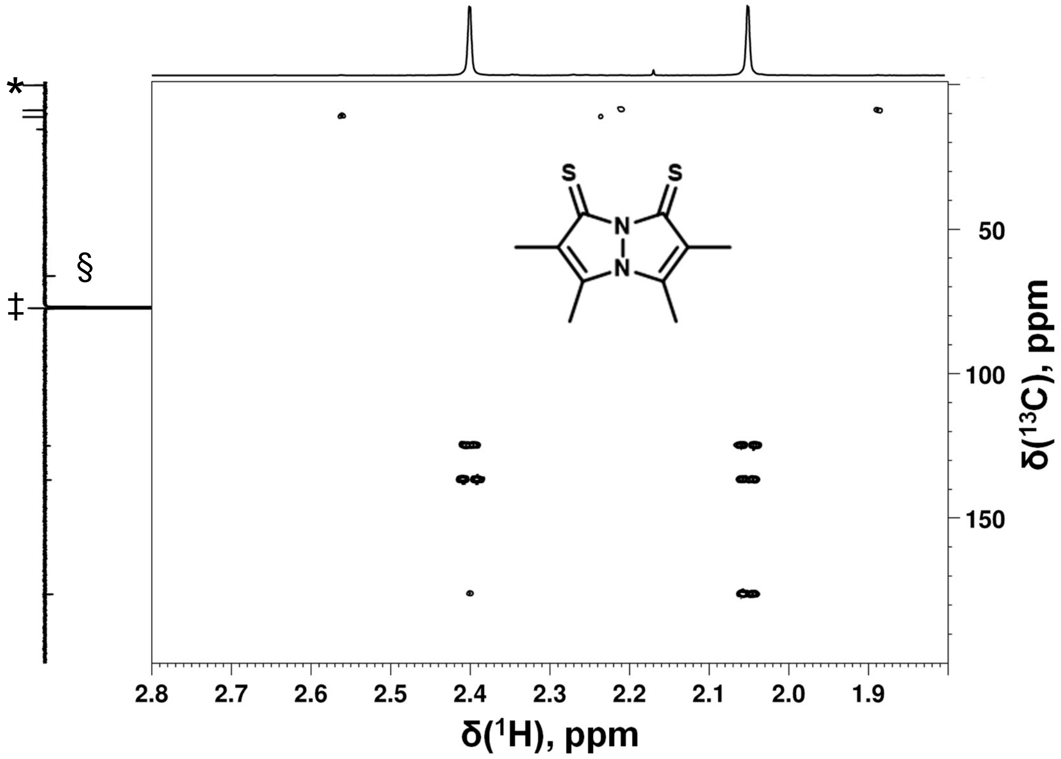

Figure S7. ^{13}C-^{1}H HMBC spectrum (101 MHz / 400 MHz) of *syn*-bimane **4** in $CDCl_3$ (*, tetramethylsilane; ‡, solvent peak; §, residual diethyl ether).

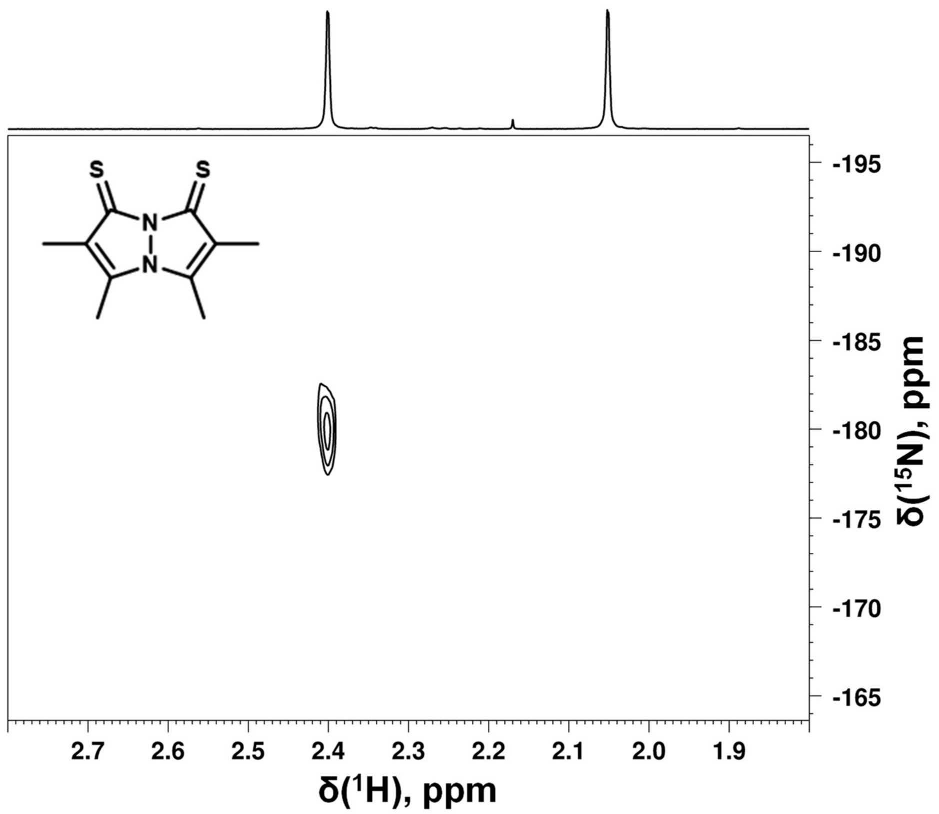

Figure S8. ^{15}N-^{1}H HMBC spectrum (41 MHz / 400 MHz) of *syn*-bimane **4** in $CDCl_3$.

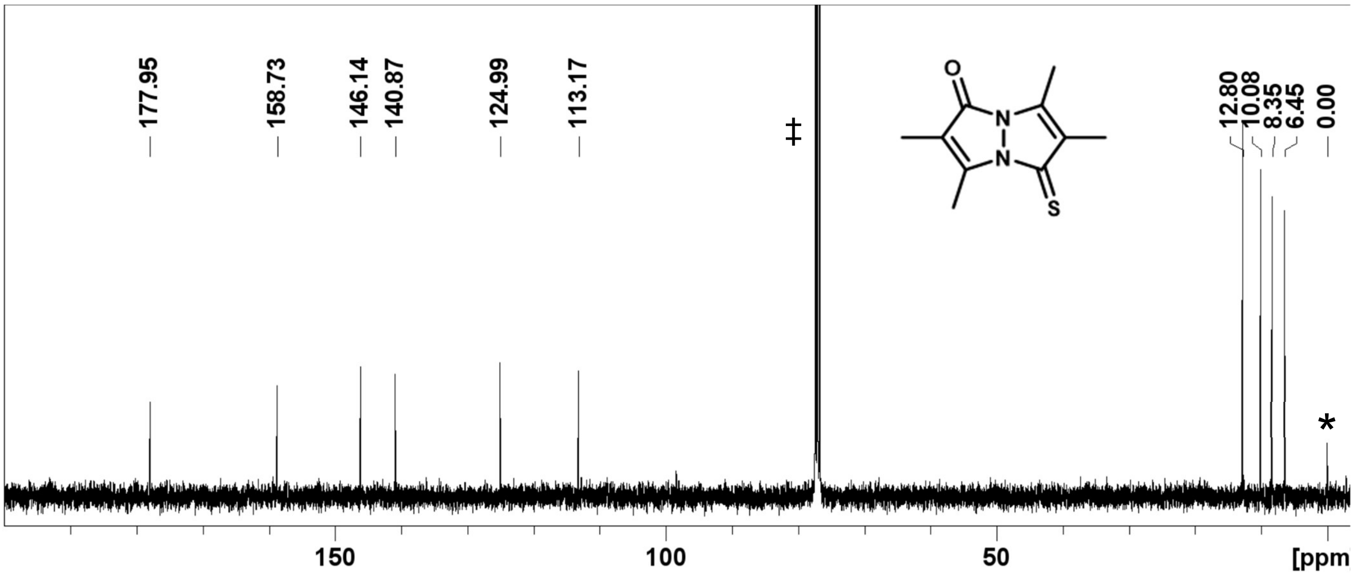

Figure S9. $^{13}C\{^{1}H\}$ NMR spectrum (101 MHz) of *anti*-bimane **5** in $CDCl_3$ (*, tetramethylsilane; ‡, solvent peak).

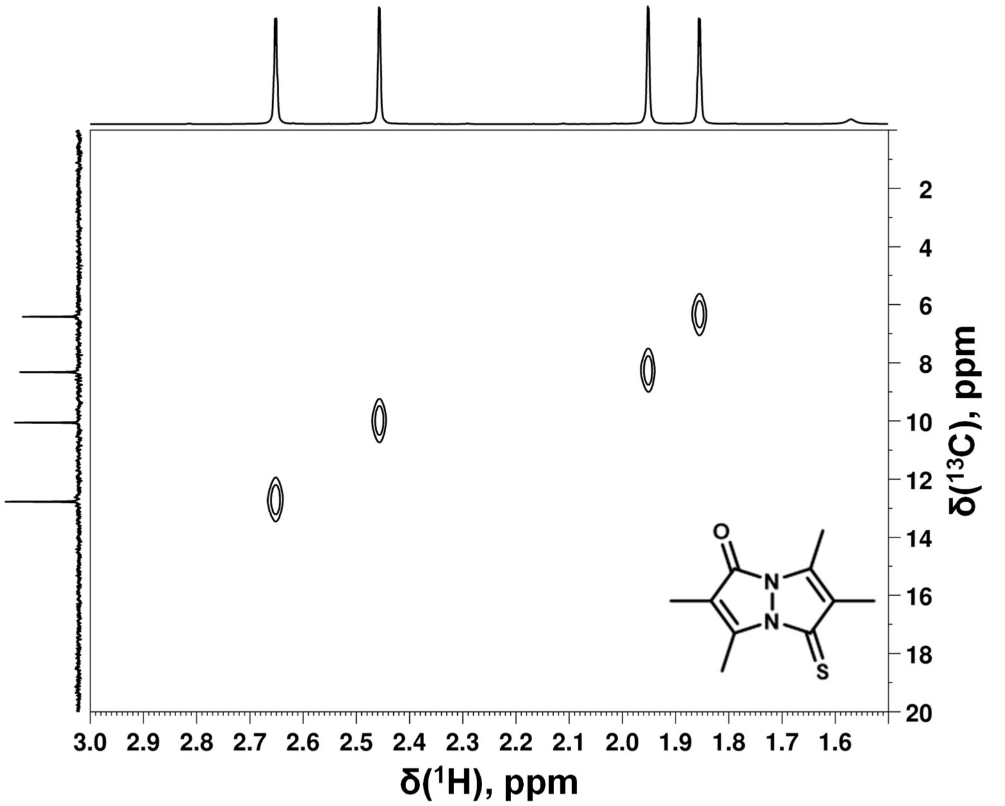

Figure S10. ^{13}C-^{1}H HMQC spectrum (101 MHz / 400 MHz) of *anti*-bimane **5** in $CDCl_3$.

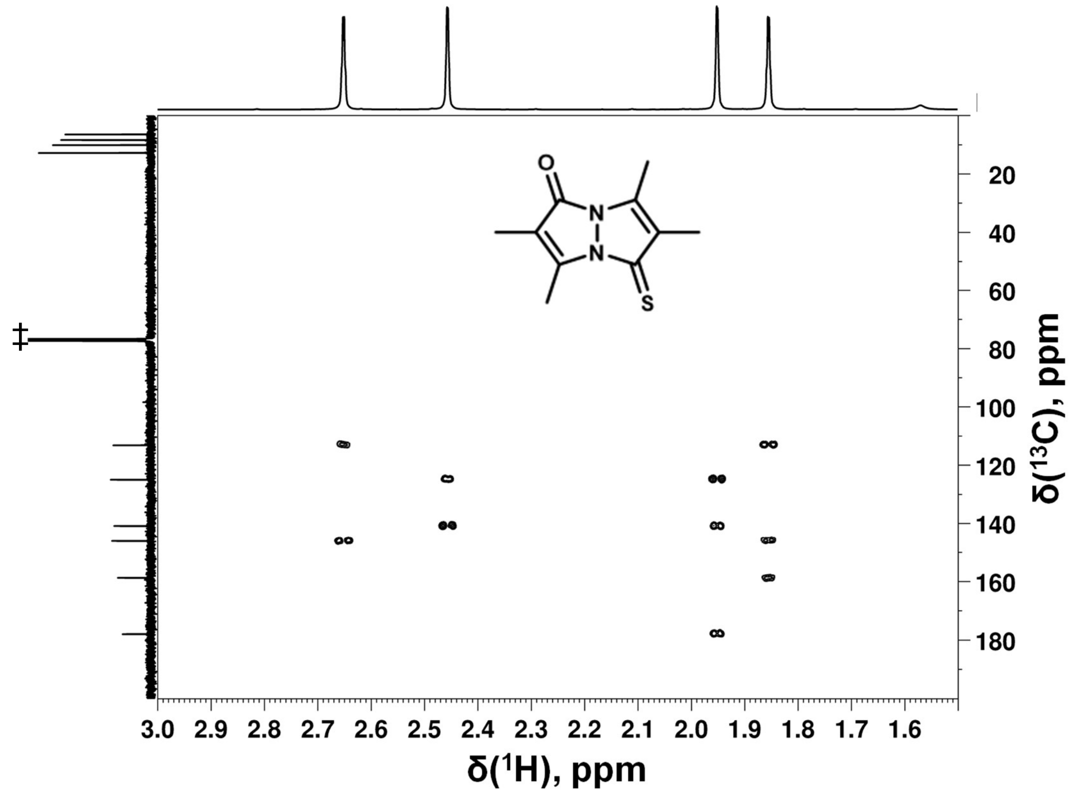

Figure S11. ^{13}C-^{1}H HMBC spectrum (101 MHz / 400 MHz) of *anti*-bimane **5** in $CDCl_3$ (‡, solvent peak).

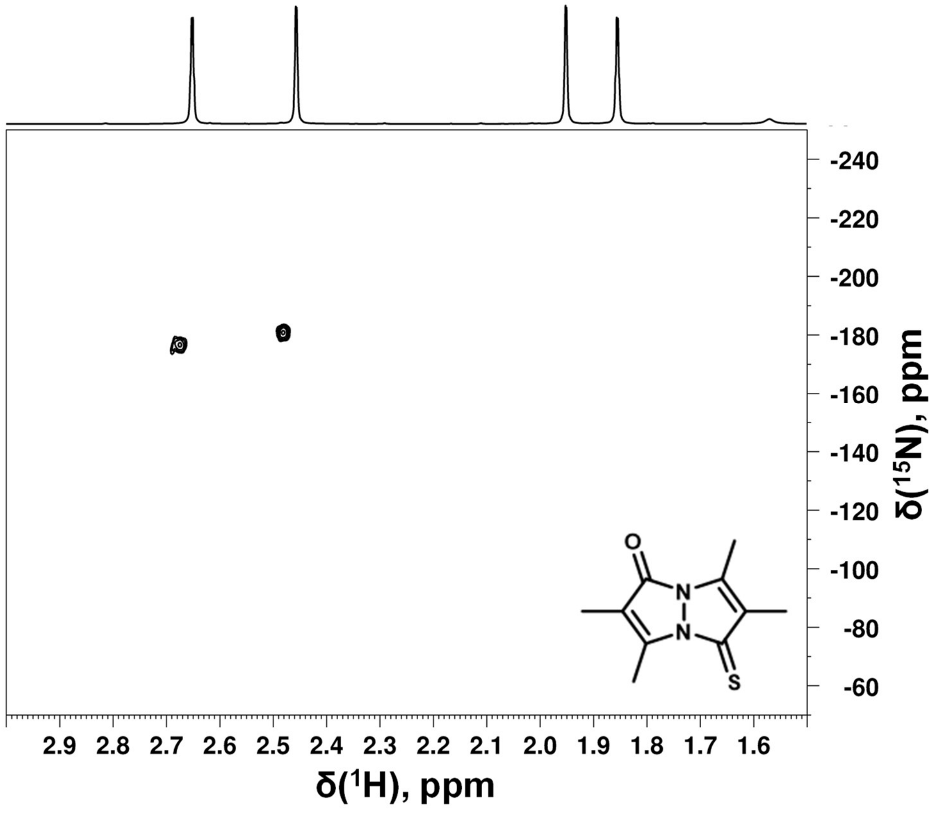

Figure S12. ^{15}N-^{1}H HMBC spectrum (41 MHz / 400 MHz) of *anti*-bimane **5** in $CDCl_3$.

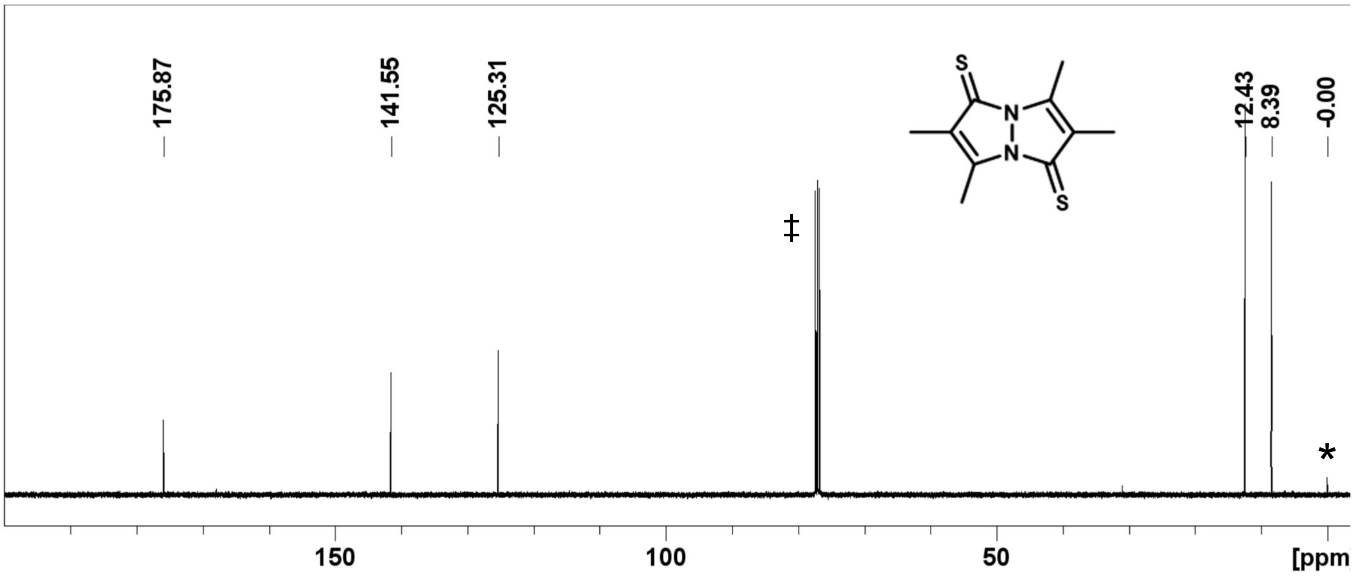

Figure S13. $^{13}C\{^{1}H\}$ NMR spectrum (101 MHz) of *anti*-bimane **6** in $CDCl_3$ (*, tetramethylsilane; ‡, solvent peak).

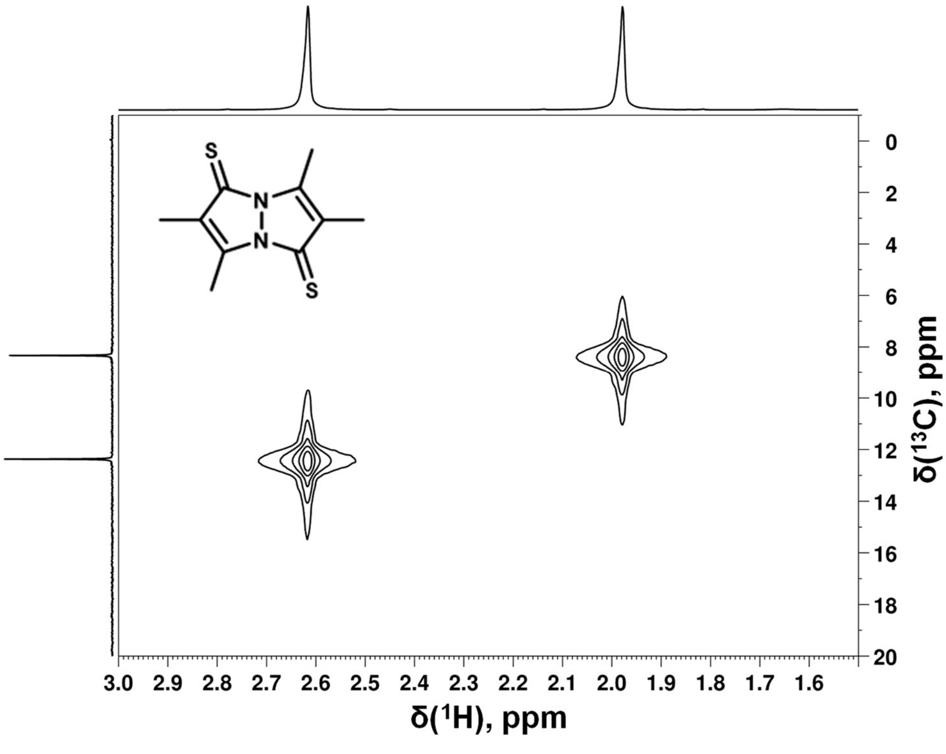

Figure S14. ^{13}C-^{1}H HMQC spectrum (101 MHz / 400 MHz) of *anti*-bimane **6** in $CDCl_3$.

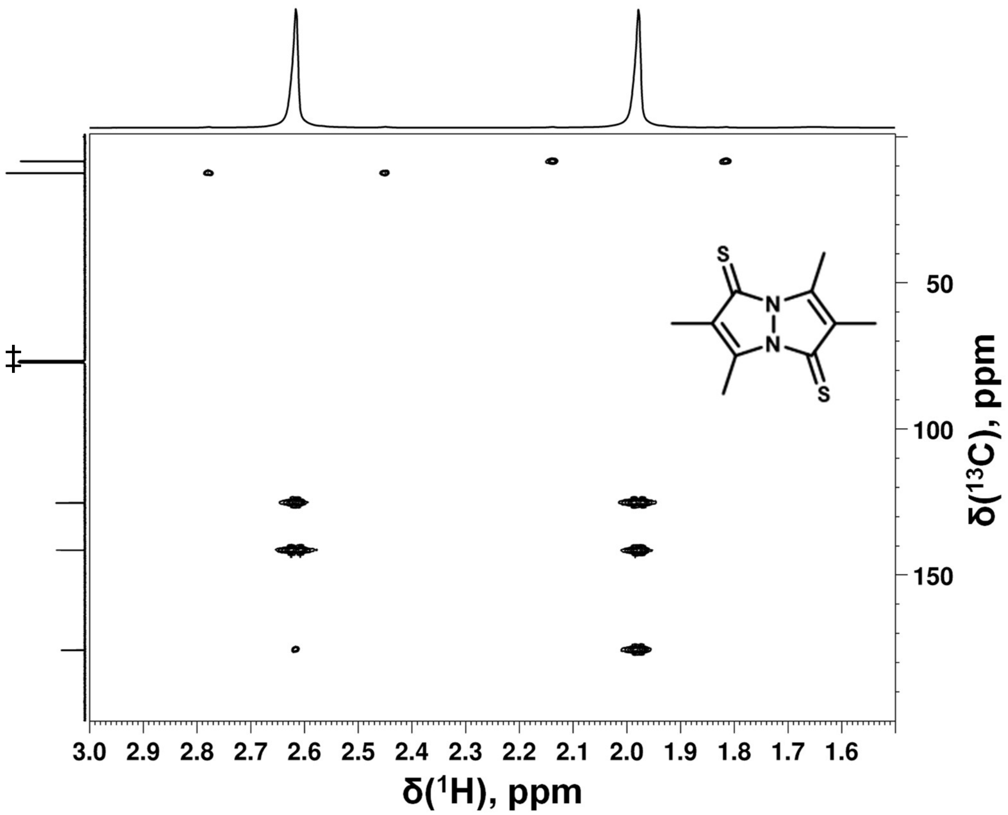

Figure S15. ^{13}C-^{1}H HMBC spectrum (101 MHz / 400 MHz) of *anti*-bimane **6** in $CDCl_3$ (‡, solvent peak).

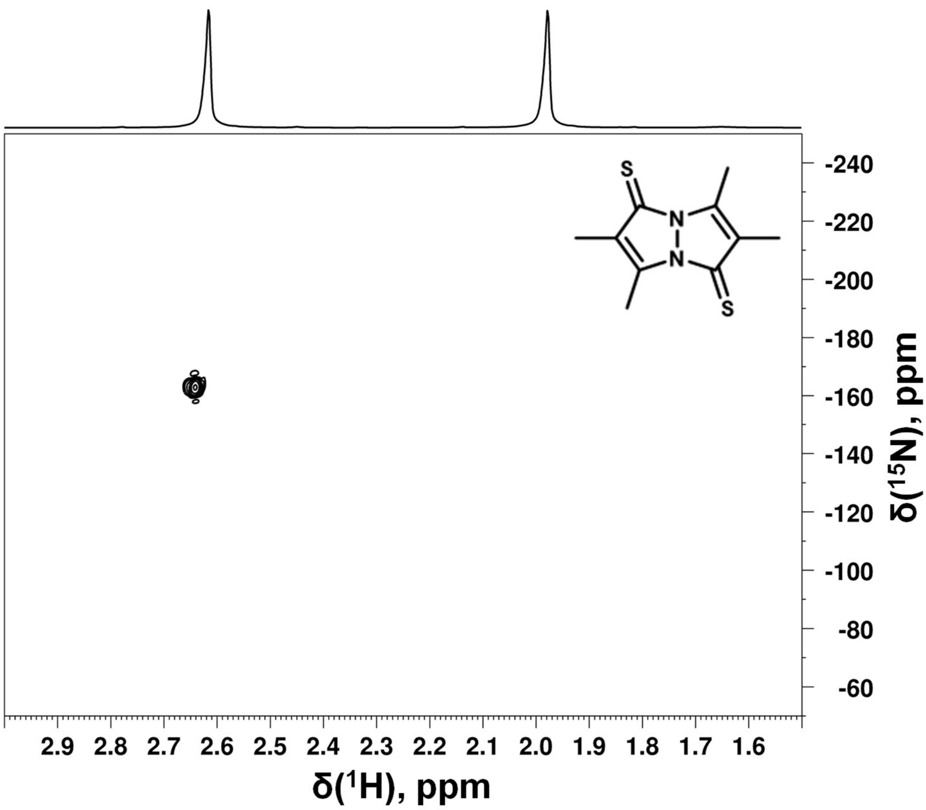

Figure S16. ^{15}N-^{1}H HMBC spectrum (41 MHz / 400 MHz) of *anti*-bimane **6** in $CDCl_3$.

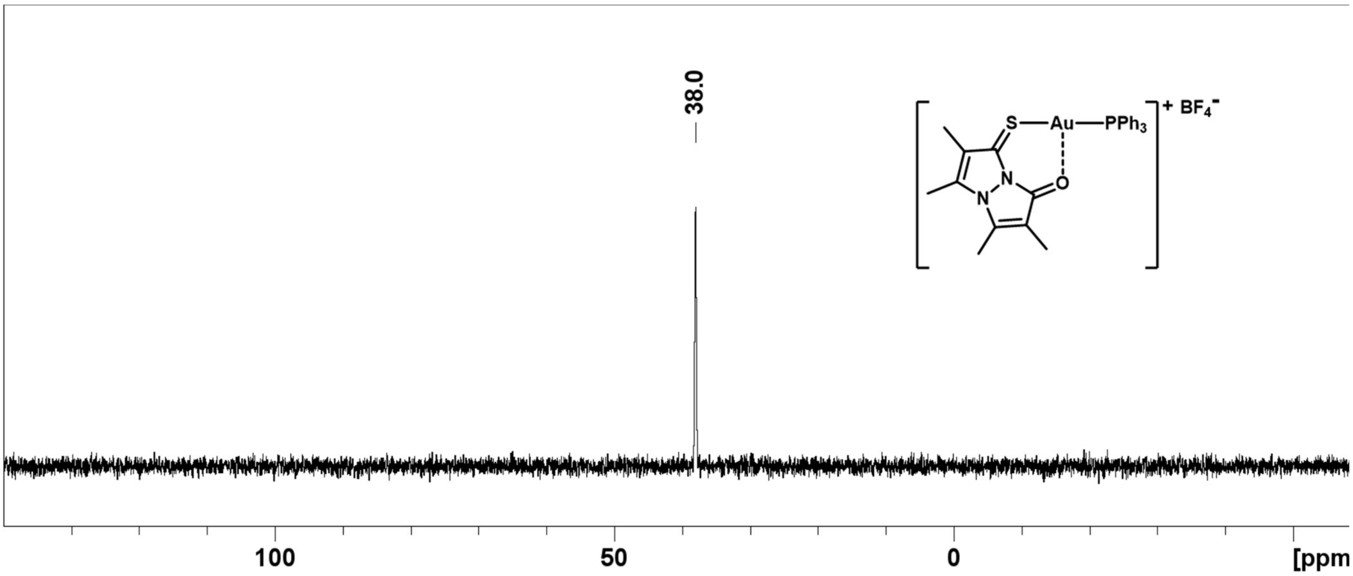

Figure S17. $^{31}P\{^{1}H\}$ NMR spectrum (162 MHz) of complex **7** in $CDCl_3$.

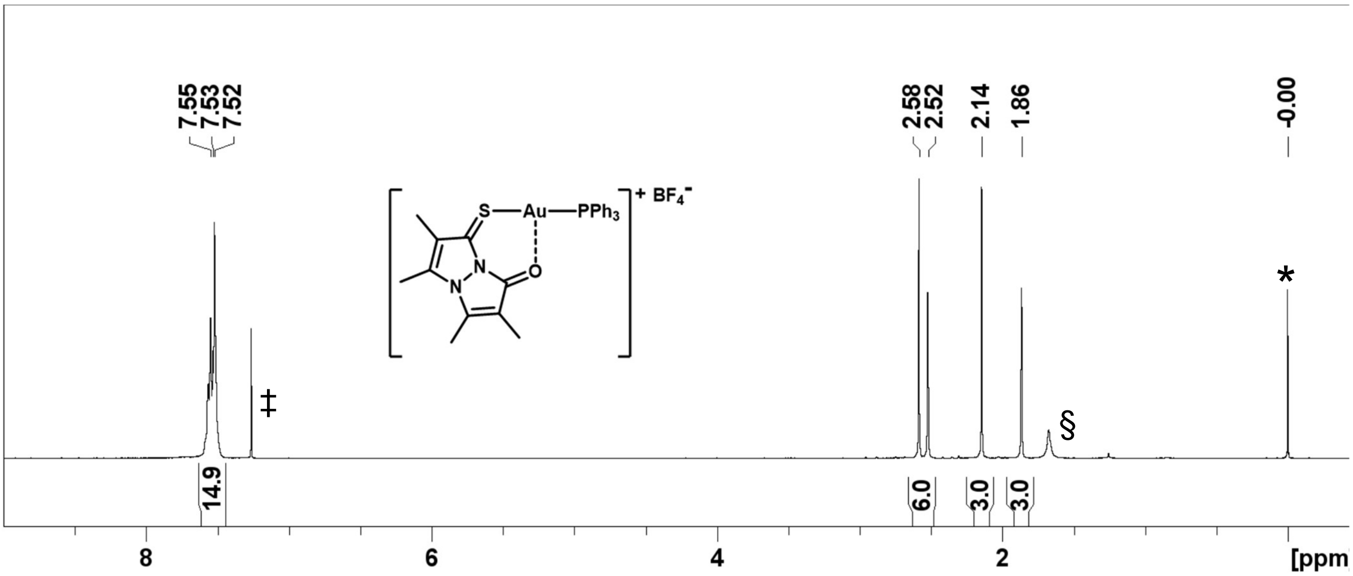

Figure S18. ^{1}H NMR spectrum (400 MHz) of complex **7** in $CDCl_3$ (*, tetramethylsilane; ‡, solvent peak; §, residual water).

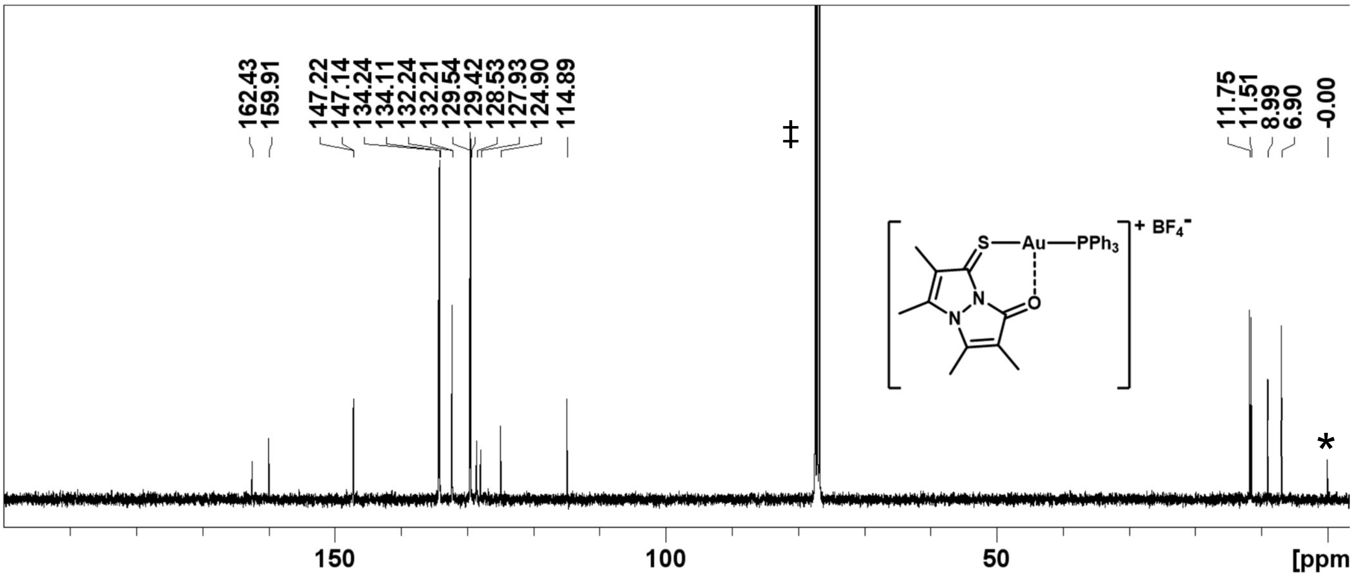

Figure S19. $^{13}C\{^1H\}$ NMR spectrum (101 MHz) of complex **7** in $CDCl_3$ (*, tetramethylsilane; ‡, solvent peak).

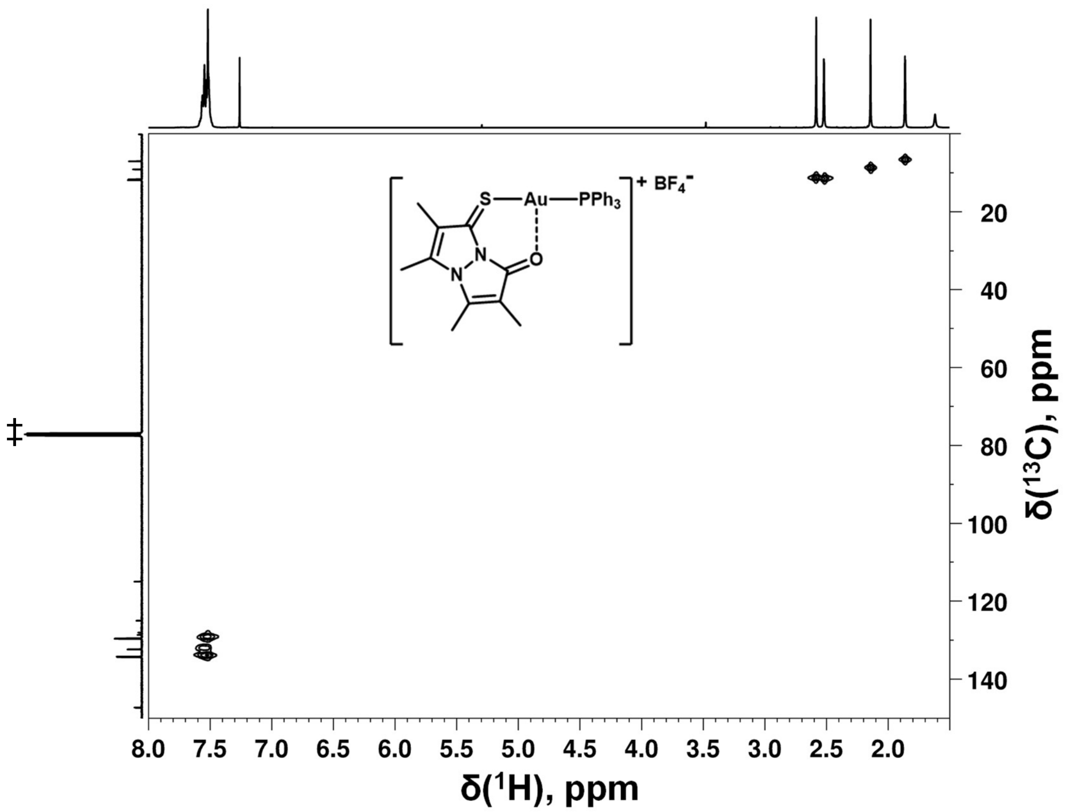

Figure S20. ^{13}C-^1H HMQC spectrum (101 MHz / 400 MHz) of complex **7** in $CDCl_3$ (‡, solvent peak).

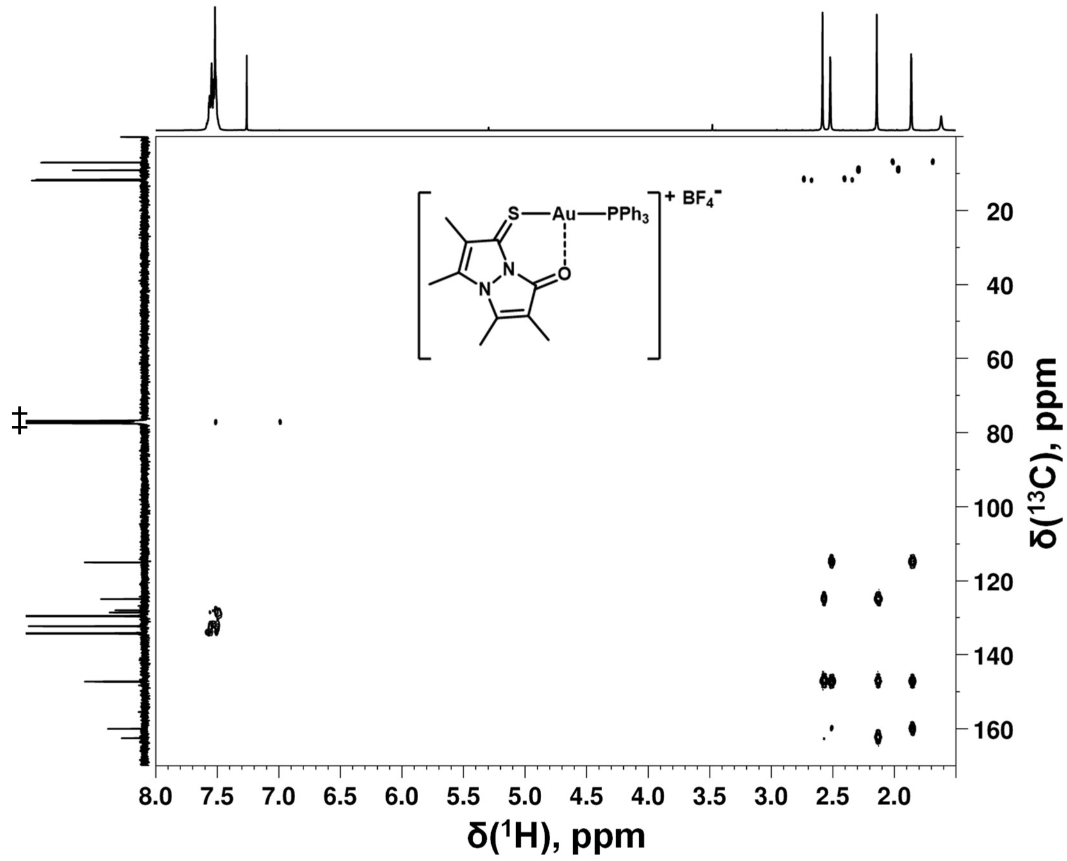

Figure S21. ^{13}C-^{1}H HMBC spectrum (101 MHz / 400 MHz) of complex **7** in $CDCl_3$ (‡, solvent peak).

Effect of oxidants on the fluorescence of solutions containing thioxobimane 4

Preliminary experiments were conducted to explore the effect of oxidants on the fluorescence of an acetonitrile solution containing 5 μM of thioxobimane **4**. Each experiment involved ~3 ml of this solution, to which an oxidant was added, as follows:

(1) NaClO: ~1 μL of commercial household bleach (~3% chlorine in water; Sano-Bruno's Enterprises Ltd, Israel).

(2) H_2O_2: ~200 μL of aqueous hydrogen peroxide (~30%).

In each case, the fluorescence spectrum of the sample was recorded before and after the addition of oxidant, with λ_{ex} = 350 nm. The results are shown in Figure S22.

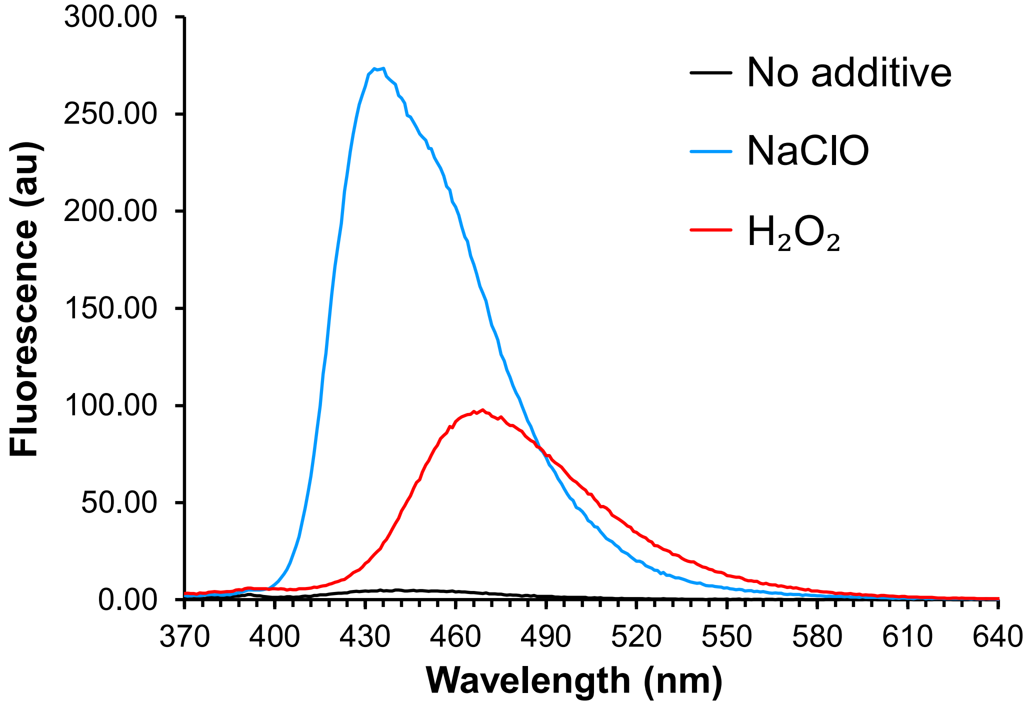

Figure S22. Fluorescence spectra of 5 μM of thioxobimane **4** in CH_3CN, before and after addition of NaClO (bleach) or hydrogen peroxide.

Table S2. Computed vibrational frequencies for bimanes 1-6 [B3LYP/6-311+G(d,p) level of theory].

Scaling Factor	0.96979
+	In-phase vibration (symmetric)
-	Out-of-phase (asymmetric)
δ	Bending
τ	Twisting
γ	Rocking
ω	wagging

Bimane 1: ***syn*****-(Me,Me)dioxobimane (C_s)**

Exp. IR ν (cm^{-1})	Calculated Scaled ν (cm-1)	ν (cm^{-1})	A^{th} ($km{\bullet}mol^{-1}$)	Raman activity	Approx. assignments
1726	1765.2	1820	764	78	ν_s (C=O)
1651	1697.1	1750	228	18	ν_{as} (C=O)
1626	1628.1	1679	62	174	ν_s (C=C)
1589	1608.0	1658	96	5	ν_{as} (C=C)
1413	1362.5	1405	190	1	ν_{as} (Me-**C–N**), ω(C-H)
1211	1188.5	1226	416	5	ν_s (O=**C–N**), ω(C-H)
	2950.0	3042	26	448	N-C-**CH_3** ν_s (C-H)
	2984.5	3077	0	144	O-C-**CH_3** ν_{as} (C-H)
	2999.9	3093	16	121	N-C-**CH_3** ν_{as} (C-H)
	2939.1	3031	0	546	O-C-**CH_3** ν_s (C-H)
	3016.3	3110	2	88	O-C-**CH_3** ν_{as} (C-H)
	3039.7	3134	1	97	N-C-**CH_3** ν_{as} (C-H)

Bimane 2: ***anti*****-(Me,Me)dioxobimane (C_1)**

Exp. IR ν (cm^{-1})	Calculated Scaled ν (cm-1)	ν (cm^{-1})	A^{th} ($km{\bullet}mol^{-1}$)	Raman activity	Approx. assignments
1678	1753.3	1807.92	1202.6934	0.4333	ν_{as} (C=O)
1624	1662.4	1714.21	199.4626	1.1055	ν_{as} (OC–**C=C**)
1265	1261.0	1300.26	408.3157	0.419	ν(N–N) - ν(O=**C–C**), δ(C-H)
	1777.7	1833.12	3.073	78.5394	ν_s (C=O)
	2986.2	3079.19	0.0611	382.4169	N-C-**CH_3** ν_s (C-H)
	3086.1	3182.25	0.7375	100.0875	N-C-**CH_3** ν_{as} (C-H)

Bimane 3: syn-(Me,Me)oxothioxobimane (C_1)

Exp. IR ν (cm^{-1})	Calculated Scaled ν (cm-1)	ν (cm^{-1})	A^{th} ($km{\bullet}mol^{-1}$)	Raman activity	Approx. assignments
1709	1732.08	1786.04	385.06	37.68	ν_s (C=O)
1622	1618.29	1668.70	72.47	201.11	ν_s (C=C)
1562	1579.82	1629.03	72.76	67.56	ν_{as} (C=C)
1412	1402.08	1445.76	268.13	6.03	ν_{as} (Me-**C–N**)*, ω(C-H)
1321	1305.39	1346.05	283.01	74.43	ν(S=**C–C**) **+** **ν**(Me-**C–N**), δ(C-H)
1277	1282.27	1322.21	79.27	14.65	ν (S=**C–N**), ω(C-H)
1248	1222.19	1260.26	348.93	13.17	ν(O=**C–N**) **+** ν(O=**C–C**), τ(C-H)
	2948.97	3040.83	29.38	545.13	N-C-**CH_3** ν_s (C-H)
	2997.63	3091.01	15.49	133.70	N-C-**CH_3** ν_{as} (C-H)

*S-containing ring

Bimane 5: ***anti*****-(Me,Me)oxothioxobimane (C_1)**

Exp. IR ν (cm^{-1})	Calculated Scaled ν (cm-1)	ν (cm^{-1})	A^{th} ($km{\bullet}mol^{-1}$)	Raman activity	Approx. assignments
1691	1708.6	1761.82	719.1487	100.8855	ν (C=O)
1626	1611.2	1661.36	114.0887	63.2249	ν (C=C)*
1595	1584.6	1633.99	62.6515	101.6733	ν (C=C)**
	1401.6	1445.22	180.6639	13.0649	ν (Me=**C–N**), ω(C-H)
	1331.4	1372.84	334.4184	15.5909	ν (S=**C–N**), ω(C-H)
	1290.4	1330.57	314.8877	75.1172	ν(S=**C–C**) **+** **ν**(Me-**C–N**), δ(C-H)
	1254.0	1293.08	269.3997	2.1995	ν(O=**C–N**) **+** ν(O=**C–C**), τ(C-H)
	2949.7	3041.62	6.1977	227.4173	N-C-**CH_3** ν_{as} (C-H)
	2984.6	3077.54	10.2961	111.736	O-C-**CH_3** ν (C-H)

*O-containing ring
**S-containing ring

Bimane 4: ***syn*****-(Me,Me)dithioxobimane (C_{2h})**

Exp. IR ν (cm^{-1})	Calculated Scaled ν (cm-1)	ν (cm^{-1})	A^{th} ($km{\bullet}mol^{-1}$)	Raman Activity	Approx. assignments
1593	1594.55	1644.22	93.49	319.95	ν_s (C=C)
1564	1572.50	1621.48	24.48	0.00	ν_{as} (C=C)
1413	1408.82	1452.71	254.17	15.86	ν_{as} (Me-**C–N**), ω(C-H)
1348	1334.79	1376.37	123.70	91.28	ν(N–N) - ν (**C=S**), δ(C-H)
1271	1258.36	1297.56	665.31	75.62	ν_{as}(S=**C–N**), δ(C-H)
	2947.91	3039.74	34.76	684.20	N-C-**CH_3** ν_s (C-H)
2852	2995.56	3088.88	16.81	150.41	N-C-**CH_3** ν_{as} (C-H)
2916	3018.36	3112.39	28.01	45.43	N-C-**CH_3** ν_{as} (C-H)

Bimane 6: ***anti*****-(Me,Me)dithioxobimane (C_{2h})**

Exp. IR ν (cm^{-1})	Calculated Scaled ν (cm-1)	ν (cm^{-1})	A^{th} ($km{\bullet}mol^{-1}$)	Raman activity	Approx. assignments
1294	1305.6	1346.32	1397.2466	0	ν_{as}(S=**C–N**), δ(C-H)
1601	1582.6	1631.93	0	156.5685	ν_s (C=C)
1416	1402.0	1445.66	124.4943	0	ν_{as} (Me-**C–N**), ω(C-H)
1203	1189.7	1226.81	104.2321	0	ν_{as} (C=S)
	1295.5	1335.89	0	227.0169	ν_s(S=**C–C**), δ(C-H)
	3040.9	3135.65	0	146.5589	N-C-**CH_3** ν_{as} (C-H)

Cartesian coordinates of the optimized geometries of bimanes 1-6 at the B3LYP/6-311+G(d,p) level of theory

Bimane 1: *syn*-(Me,Me)dioxobimane (C_s)

O	-2.442542	0.002027	1.643068
O	-2.442542	0.002027	-1.643068
N	-0.806489	-0.361914	0.000000
N	0.580459	-0.354863	0.000000
C	-1.281567	-0.073996	1.316228
C	-0.057115	0.072937	2.117754
C	1.019473	-0.058959	1.297297
C	1.019473	-0.058959	-1.297297
C	-0.057115	0.072937	-2.117754
C	-1.281567	-0.073996	-1.316228
C	-0.118849	0.298901	3.592638
C	2.474499	-0.006627	1.626752
C	2.474499	-0.006627	-1.626752
C	-0.118849	0.298901	-3.592638
H	-0.673429	1.214207	3.818112
H	0.873584	0.383692	4.037989
H	-0.649458	-0.519775	4.088011
H	2.605752	0.066875	2.705379
H	2.963947	0.858997	1.172054
H	2.992401	-0.905024	1.281726
H	2.605752	0.066875	-2.705379
H	2.992401	-0.905024	-1.281726
H	2.963947	0.858997	-1.172054
H	-0.673429	1.214207	-3.818112
H	-0.649458	-0.519775	-4.088011
H	0.873584	0.383692	-4.037989

Bimane 2: *anti*-(Me,Me)dioxobimane (C_1)

O	1.340435	-2.491082	0.071753
N	-0.074737	-0.686438	-0.393831
N	0.074743	0.686459	-0.393789
C	1.170270	-1.294594	-0.055698
C	2.103959	-0.169128	0.065273
C	1.406658	0.989703	-0.098053
C	-1.170264	1.294596	-0.055621
C	-2.103960	0.169124	0.065249
C	-1.406654	-0.989697	-0.098118
C	3.559399	-0.378751	0.329643
C	1.836910	2.414001	-0.052641
C	-3.559408	0.378733	0.329585

H	3.705303	-0.981642	1.230178
H	4.086322	0.567389	0.461476
H	4.033000	-0.920649	-0.494801
H	2.869586	2.490744	0.285269
H	1.189718	2.989493	0.613311
H	1.754378	2.870800	-1.043330
H	-4.086337	-0.567415	0.461337
H	-4.032982	0.920689	-0.494837
H	-3.705339	0.981564	1.230156
C	-1.836907	-2.413998	-0.052792
H	-1.754363	-2.870740	-1.043506
H	-1.189724	-2.989529	0.613136
H	-2.869588	-2.490760	0.285100
O	-1.340440	2.491079	0.071856

Bimane 3: syn-(Me,Me)oxothioxobimane (C_1)

S	-2.334978	-2.031837	-0.002116
O	1.075868	-2.631316	0.011890
N	-0.042938	-0.576531	-0.221113
N	0.366309	0.739488	-0.219420
C	-1.427478	-0.650691	-0.052584
C	-1.855694	0.736550	0.044372
C	-0.752502	1.543797	-0.032517
C	1.752090	0.784951	-0.036145
C	2.227468	-0.487761	0.047088
C	1.098350	-1.425531	-0.039906
C	-3.289389	1.130213	0.180695
H	-3.412460	2.213235	0.229107
H	-3.723093	0.691914	1.083495
H	-3.874292	0.750886	-0.661845
C	-0.648655	3.031909	0.005381
H	-0.091832	3.377722	0.880761
H	-1.644696	3.469014	0.051285
H	-0.149545	3.421414	-0.885834
C	2.486958	2.083234	-0.011304
H	2.290000	2.671219	-0.911589
H	3.558602	1.897810	0.042750
H	2.208352	2.690543	0.854175
C	3.631418	-0.979551	0.178253
H	3.738083	-1.604796	1.069173
H	4.348100	-0.160033	0.249147
H	3.906491	-1.602444	-0.678167

Bimane 4: ***syn*-(Me,Me)dithioxobimane (C_{2h})**

N	0.000000	0.000000	-0.359575
N	0.000000	0.000000	1.013300
C	0.000000	1.320137	-0.833349
C	0.000000	2.136197	0.369847
C	0.000000	1.314002	1.464616
C	0.000000	-1.314002	1.464616
C	0.000000	-2.136197	0.369847
C	0.000000	-1.320137	-0.833349
C	0.000000	3.628855	0.330943
H	0.876637	4.000094	-0.206684
H	0.000000	4.061234	1.332637
H	-0.876637	4.000094	-0.206684
C	0.000000	1.643133	2.919621
H	0.000000	2.723889	3.050490
H	0.884108	1.244065	3.424366
H	-0.884108	1.244065	3.424366
C	0.000000	-1.643133	2.919621
H	0.884108	-1.244065	3.424366
H	0.000000	-2.723889	3.050490
H	-0.884108	-1.244065	3.424366
C	0.000000	-3.628855	0.330943
H	-0.876637	-4.000094	-0.206684
H	0.000000	-4.061234	1.332637
H	0.876637	-4.000094	-0.206684
S	0.000000	1.803306	-2.413538
S	0.000000	-1.803306	-2.413538

Bimane 5: ***anti*-(Me,Me)oxothioxobimane (C_1)**

O	-2.200815	2.206991	0.039875
N	-0.295483	0.872794	-0.167373
N	-0.043726	-0.474572	-0.161586
C	-1.687980	1.107304	-0.018335
C	-2.269182	-0.240891	0.026899
C	-1.263290	-1.158369	-0.041135
C	1.322241	-0.701503	-0.032399
C	1.910967	0.629456	0.024389
C	0.904675	1.555828	-0.038192
C	-3.744653	-0.451017	0.128600
C	-1.303847	-2.644066	-0.023800
C	3.382225	0.862745	0.127014
H	-4.145888	0.045984	1.016367
H	-3.999706	-1.509780	0.185669
H	-4.260257	-0.021275	-0.735465

H	-2.334679	-2.986752	0.060715
H	-0.719757	-3.040021	0.810606
H	-0.859403	-3.057332	-0.932514
H	3.616102	1.927178	0.176232
H	3.903697	0.433467	-0.733087
H	3.789684	0.375765	1.016934
C	0.915128	3.044222	-0.017931
H	0.548758	3.446860	-0.966537
H	0.252554	3.425639	0.762775
H	1.924565	3.414304	0.155114
S	2.079226	-2.182486	0.022265

Bimane 6: *anti*-(Me,Me)dithioxobimane (C_{2h})

N	-0.004978	0.684781	0.000000
N	0.004978	-0.684781	0.000000
C	-1.313785	1.157597	0.000000
C	-2.133413	-0.044011	0.000000
C	-1.313785	-1.140362	0.000000
C	1.313785	-1.157597	0.000000
C	2.133413	0.044011	0.000000
C	1.313785	1.140362	0.000000
C	-3.625669	-0.002852	0.000000
C	-1.627028	-2.592692	0.000000
C	3.625669	0.002852	0.000000
H	-3.997890	0.532993	0.877442
H	-4.055589	-1.005147	0.000000
H	-3.997890	0.532993	-0.877442
H	-2.706547	-2.739560	0.000000
H	-1.196587	-3.086547	0.874798
H	-1.196587	-3.086547	-0.874798
H	4.055589	1.005147	0.000000
H	3.997890	-0.532993	-0.877442
H	3.997890	-0.532993	0.877442
C	1.627028	2.592692	0.000000
H	1.196587	3.086547	-0.874798
H	1.196587	3.086547	0.874798
H	2.706547	2.739560	0.000000
S	1.785174	-2.751001	0.000000
S	-1.785174	2.751001	0.000000
